# Cardioprotective potential of oleuropein, hydroxytyrosol, oleocanthal and their combination: Unravelling complementary effects on acute myocardial infarction and metabolic syndrome

**DOI:** 10.1016/j.redox.2024.103311

**Published:** 2024-08-14

**Authors:** Andriana Christodoulou, Panagiota-Efstathia Nikolaou, Lydia Symeonidi, Konstantinos Katogiannis, Louisa Pechlivani, Theodora Nikou, Aimilia Varela, Christina Chania, Stelios Zerikiotis, Panagiotis Efentakis, Dimitris Vlachodimitropoulos, Nikolaos Katsoulas, Anna Agapaki, Costantinos Dimitriou, Maria Tsoumani, Nikolaos Kostomitsopoulos, Constantinos H. Davos, Alexios Leandros Skaltsounis, Alexandros Tselepis, Maria Halabalaki, Ioulia Tseti, Efstathios K. Iliodromitis, Ignatios Ikonomidis, Ioanna Andreadou

**Affiliations:** a Laboratory of Pharmacology, Faculty of Pharmacy, National and Kapodistrian University of Athens, Panepistimioupolis, Zografou, Athens, Greece; b Laboratory of Echocardiography and Preventive Cardiology, Second Cardiology Department, Attikon University Hospital, National and Kapodistrian University of Athens, Medical School, Athens, Greece; c Atherothrombosis Research Centre/Laboratory of Biochemistry, Department of Chemistry, University of Ioannina, Ioannina, Greece; d Division of Pharmacognosy and Natural Products Chemistry, Department of Pharmacy, National and Kapodistrian University of Athens, Athens, Greece; e Cardiovascular Research Laboratory, Biomedical Research Foundation Academy of Athens (BRFAA), Athens, Greece; f Laboratory of Forensic Medicine and Toxicology, Medical School National and Kapodistrian University of Athens, Athens, Greece; g Histochemistry Unit, Biomedical Research Foundation, Academy of Athens (BRFAA), Athens, Greece; h Centre of Clinical Experimental Surgery and Translational Research, Biomedical Research Foundation of the Academy of Athens (BRFAA), Athens, Greece; i Uni-Pharma S.A., Athens, Greece; j National and Kapodistrian University of Athens, Medical School, Athens, Greece

**Keywords:** Cardiometabolic syndrome, Ischemia-reperfusion injury, Oleuropein, Hydroxytyrosol, Oleocanthal, Oleanolic acid

## Abstract

Clinical studies have previously established the role of olive products in cardiovascular disease (CVD) prevention, whilst the identification of the responsible constituents for the beneficial effects is still pending. We sought to assess and compare the cardioprotective potential of oleuropein (OL), hydroxytyrosol (HT), oleocanthal (OC) and oleanolic Acid (OA), regarding Ischemia/Reperfusion Injury (IRI) and CVD risk factors alleviation. The scope of the study was to design a potent and safe combinatorial therapy for high-cardiovascular-risk patients on a bench-to-bedside approach. We evaluated the IRI-limiting potential of 6-weeks treatment with OL, HT, OC or OA at nutritional doses, in healthy and metabolic syndrome (MS)-burdened mice. Three combinatorial regimens were designed and the mixture with preponderant benefits (OL-HT-OC, Combo 2), including infarct sparing and antiglycemic potency, compared to the isolated compounds, was further investigated for its anti-atherosclerotic effects. *In vivo* experiments revealed that the combination regimen of Combo 2 presented the most favorable effects in limiting infarct size and hyperglycemia, which was selected to be further investigated in the clinical setting in Chronic Coronary Artery Syndrome (CCAS) patients. Cardiac function, inflammation markers and oxidative stress were assessed at baseline and after 4 weeks of treatment with the OL-HT-OC supplement in the clinical study. We found that OL, OC and OA significantly reduced infarct size *in vivo* compared to Controls. OL exhibited antihyperglycemic properties and OA attenuated hypercholesterolemia. OL-HT-OA, OL-HT-OC and OL-HT-OC-OA combination regimens were cardioprotective, whereas only OL-HT-OC mitigated hyperglycemia. Combo 2 cardioprotection was attributed to apoptosis suppression, enhanced antioxidant effects and upregulation of antioxidant enzymes. Additionally, it reduced atherosclerotic plaque extent *in vivo*. OL-HT-OC supplement ameliorated cardiac, vascular and endothelial function in the small-scale clinical study. Conclusively, OL-HT-OC combination therapy exerts potent cardioprotective, antihyperglycemic and anti-atherosclerotic properties *in vivo*, with remarkable and clinically translatable cardiovascular benefits in high-risk patients.

## Highlights

• Oleuropein, oleocanthal and oleanolic acid limit infarct size at nutritional doses.
• The combination of hydroxytyrosol, oleuropein and oleocanthal (Combo 2) is cardioprotective in presence of metabolic syndrome.
• Combo 2 attenuates hyperglycemia, LDL oxidation and atherosclerosis development.
• Combo 2 exerts antioxidant and antiapoptotic properties in the ischemic heart.
• Combo 2-based supplement improves cardiovascular function in CCAS patients.

## Graphical abstract

**Figure:**
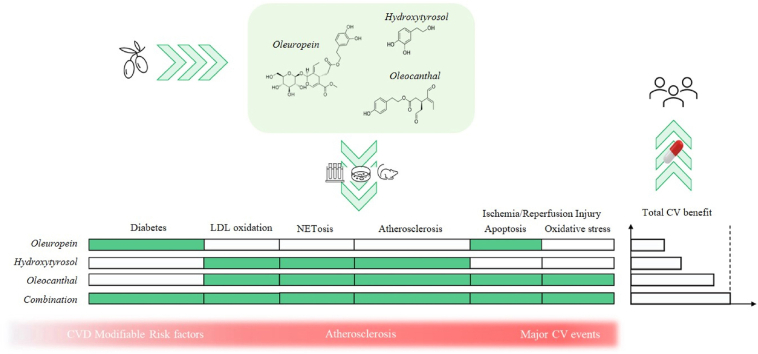

## Abbreviations

3-NT: 3- Nitrotyrosine
Α: late mitral inflow velocity
Aix: Augmentation Index
Akt: Protein Kinase B
ALP: Alkaline Phosphatase
ALT: Alanine Aminotransferase
ANOVA: Analysis of variance
AST: Aspartic Aminotransferase
AUC: Area Under Curve
BcL-xL: B-cell lymphoma-extra large
brDBP: brachial Diastolic Blood Pressure
brSBP: brachial Systolic Blood Pressure
CAT: Catalase
CCAS: Chronic Coronary Artery Syndrome
cDBP: central Diastolic Blood Pressure
CFR: Coronary Flow Reserve
cPP: central pulse pressure
CRP: C-Reactive Protein
cSBP: central Systolic Blood Pressure
CVD: cardiovascular disease
DAPI: 49,6‐diamidino‐2‐phenylindole
DHE: dihydroethidium
DMSO: Dimethyl-sulfoxide
DT: Deceleration Time
E: early mitral inflow velocity by Doppler
e’: average lateral and septal velocity of mitral annulus
ECG: electrocardiogram
EF: Ejection Fraction
EVOO: Extra Virgin Olive Oil
FCPC: Fast Centrifugal Partition Chromatography
FCPE: Fast Centrifugal Partition Extractor
FMD: Flow-Mediated Dilation
FS: Fractional Shortening
GAPDH: Glyceraldehyde-3-phosphate dehydrogenase
gp91-phox: heme binding subunit of the superoxide-generating NADPH oxidase
GSK-3β: Glycogen Synthase Kinase 3 beta
GTT: Glucose Tolerance Test
H&E: Hematoxylin and Eosin
HO-1: Heme-oxygenase-1
HOMAIR: Homeostatic Model Assessment for Insulin Resistance
HPLC-DAD: High-Performance Liquid Chromatography with Diode-Array Detection
HT: Hydroxytyrosol
IRI: Ischemia/Reperfusion Injury
IS: Infarct Size
IVSd: Interventricular septum thickness at end diastole
LA ESV BP: Left Atrial End-Systolic Volume
LAD: Left Anterior Descending Artery
LAd: Left Atrial Diameter
LC-HRMS: Liquid ChromatographyHigh Resolution Mass Spectrometry
LDH: Lactic Dehydrogenase
Lp(a): Lipoprotein-a
LV: Left Ventricular
LVEDD: Left Ventricular End-Diastolic Diameter
LVEDV: Left Ventricular End Diastolic Volume
LVESD: Left Ventricular End-Systolic Diameter
LVESV: Left Ventricular End Systolic Volume
LVGLS: Left Ventricular Global longitudinal strain
LVMi: Left Ventricular Mass index
MDA: Malondialdehyde
MnSOD: Manganese-superoxide dismutase
MPO: Myeloperoxidase
MS: Metabolic Syndrome
ND: Normal Diet- Standard Chow Diet
NET: Neutrophil Extracellular Trap
NMR: Nuclear Μagnetic Resonance
NOX-1: NADPH oxidase 1
Nrf2: Nuclear factor erythroid 2–related factor 2
NS: Normal Saline
OA: Oleanolic Acid
OC: Oleocanthal
OL: Oleuropein
OxLDL: Oxidized Low-Density Lipoprotein
PBR: Perfused Boundary Region
p47^phox^: NADPH oxidase cytosolic protein p47phox
PCI: Percutaneous Coronary Intervention
PCSK9: Proprotein Convertase Subtilisin/Kexin Type 9
PMA: Phorbol 12-myristate 13-acetate
PWd: Posterior wall thickness at end diastole
PWTd: Left Ventricular posterior wall thickness at diastole
PWTs: Left Ventricular posterior wall thickness at systole
PWV: Pulse Wave Velocity
r/h: Left Ventricular radius to Posterior Wall Thickness ratio
RISK: Reperfusion Injury Salvage Kinase
ROS: Reactive Oxygen Species
RPMI: Roswell Park Memorial Institute
RWT: Relative Wall Thickness
SD: Standard Deviation
SEM: Standard Error of Means
TDI: Tissue Doppler Imaging
TLC: Thin Layer Chromatography
TPF: Total Phenolic Fraction
TTC: 2,3,5-Triphenyltetrazolium chloride
WD: Western Diet

## 1 Introduction

Cardiovascular diseases (CVDs) present a significant public health challenge, as they remain the leading cause of morbidity and mortality worldwide [1]. In acute coronary syndromes, cardioprotection in terms of infarct size reduction is pivotal for improving clinical outcomes and mortality. However, numerous cardioprotective approaches have failed to mitigate Ischemia-Reperfusion Injury (IRI) in presence of confounding pathologies such as obesity, insulin resistance, atherogenic dyslipidemia, the coexistence of which is characterized as metabolic syndrome (MS) [[2], [3], [4], [5]]. Cardioprotective strategies emphasize the use of multifaceted interventions to achieve maximum effectiveness. However, there is currently no approved pharmacological intervention to reduce ischemia-reperfusion injury (IRI) in the presence or absence of metabolic syndrome (MS) [6].

It is generally appreciated that the effective management of MS requires lifestyle modifications [7,8]. Clinical trials demonstrate that extra virgin olive oil (EVOO) consumption is interlinked with enhanced glucose homeostasis [9,10], improved lipid metabolism [11], reduced blood pressure [12], while several reports support its antiatherosclerotic [13], antioxidant [14,15] and anti-inflammatory effects [11,16,17], in diverse populations at high risk of developing CVDs. These beneficial effects are mostly attributed to the polar or hydrophilic olive-derived compounds usually referred as olive biophenols. Hydroxytyrosol (HT) and its derivatives have obtained a health claim by the European Food Safety Authority (EFSA) for their protective effect against Low Density Lipoprotein (LDL) oxidative modifications [18]. HT has shown a positive influence on various parameters of MS including obesity, hypercholesterolemia and insulin resistance *in vivo* [[19], [20], [21], [22]]. The secoiridoid oleuropein (OL) has antihypertensive [23], hypoglycemic [24,25], antioxidant [26], hypolipidemic [26] and antiobesity properties [27]. OL is cardioprotective after acute [28] and chronic [26] administration and also against doxorubicin-induced cardiotoxicity [29,30] *in vivo*. Oleocanthal (OC) has anti-inflammatory properties [31,32]. Oleanolic acid (OA) protects the heart against ischemia/reperfusion [33] and isoproterenol-induced injury [34], while antihypertensive [35], antidiabetic, anti-inflammatory, antioxidant and hypolipidemic [36,37] effects have also been reported. However, the cardioprotective effect of the above-mentioned bioactive constituents against IRI in the presence of comorbidities remains to be elucidated especially at nutritional doses, corresponding to the human intake according to the Mediterranean diet.

Based on the above, we hypothesized that the possible additive effect among the constituents of olive oil, could be implemented for the discovery of combinatorial treatments against CVDs in terms of cardioprotection and risk factors mitigation. We aimed to explore the cardioprotective effects of the four isolated compounds OL, HT, OC and OA after chronic administration, at nutritional doses, in a murine *in vivo* model of MS and IRI. Consequently, we sought to design three combinatorial treatments in a multi-targeted strategy approach, to: 1) alleviate MS progression as a cardiovascular disease risk factor and 2) mitigate the myocardial damage in case of major coronary events. Moreover, we aimed to investigate the cardioprotective and anti-atherosclerotic effects of the combinatorial treatment and its constituents, with a particular emphasis on oxidative stress aspects. The most promising combination was further evaluated in a proof-of-concept clinical study in chronic coronary artery syndrome (CCAS) patients. Our ultimate aim was the successful application of nutraceuticals’ vast biological activities in the clinical practice to comprehensively shield patients at high cardiovascular risk, in terms of improved myocardial, arterial and endothelial function.

## 2 Materials and methods

For complete Methods please refer to the Supplementary Material.

All reagents were purchased from Panreac AppliChem unless otherwise stated. Antibodies were purchased from Cell Signaling Technology (distributor Bioline, E. Demagkos & Co, Athens, Greece), unless otherwise stated.

### 2.1 Compounds isolation, purification and identification

The isolation and purification of the target compounds i.e. HT (PubChem CID 82755), OC (PubChem CID 11652416) and OA (PubChem CID 10494), was performed using EVOO as starting material (2019–2020 harvesting period), while for OL (PubChem CID 5281544) olive leaves were used (*Olea* europea var. Koronoiki). More specifically, for the isolation and purification of the HT, OC and OA a previously established workflow was followed combining two solid-free liquid-liquid chromatographic techniques i.e. a Fast Centrifugal Partition Extractor (FCPE)-based extraction and a Fast Centrifugal Partition Chromatograph (FCPC)-based fractionation, as well as a final purification step using column chromatography [38]. Initially an FCPE-based extraction process was employed for the recovery of biophenols from EVOO or Total Phenolic Fraction (TPF). Afterwards, TPF was subjected to stepwise gradient FCPC fractionation using four different biphasic systems. Finally, the obtained fractions containing the target compounds, were further purified using size exclusion (sephadex) and adsorption chromatography (silica gel). Regarding OL, a divergent procedure was followed with some modifications, due to the different starting material, namely olive leaves [39]. In this case and in line with the previous isolation protocols, FCPC technique was employed as a key chromatographic technique. Thin Layer Chromatography – TLC was used to monitor qualitatively the entire procedures. For structure verification, Liquid Chromatography-High Resolution Mass Spectrometry (LC-HRMS) and Nuclear magnetic resonance (NMR) were used, while High-Performance Liquid Chromatography with Diode-Array Detection (HPLC-DAD) was employed for purity determination (Supplementary Fig. 1E). Finally, OL (98 % purity, HPLC-DAD) (Supplementary Fig. 1A), HT (98.5 %, HPLC-DAD) (Supplementary Fig. 1B), OC (98.5 % purity, HPLC-DAD) (Supplementary Fig. 1C) and OA (98 %, NMR) (Supplementary Fig. 1D) were obtained. The isolation and purification procedures were repeated to acquire the required quantity of each compound for the *in vitro* and the in *vivo* experiments. Detailed procedures are presented in the Supplementary Material.

While OL and HT treatment solutions were prepared in sterile normal saline (NaCl 0.9 %) (Normal Saline, NS), OC and OA solutions were prepared in 5 % v/v Dimethyl-sulfoxide (DMSO) in NS due to their low water-solubility [40,41]. Therefore, results for each group were compared to the respective vehicle group, namely Vehicle (NS) for OL and HT and Vehicle (DMSO 5 %) for OC and OA.

### 2.2 Dose selection

Dose selection was based on the nutritional human intake following the Mediterranean diet to maintain high translational value. For OL and HT, we used doses referred as nutritional or based on the human daily intake of these compounds during the Mediterranean diet in previous *in vivo* studies [22,26,42]. However, due to vast variations of OC and OA concentrations in olive products, estimation of the nutritionally equivalent doses was challenging. Hence, we calculated and used the equimolar to OL (20.6 mg/kg) and HT (5.9 mg/kg) doses for OC (11.6 mg/kg) and OA (17.4 mg/kg), with the concomitant benefit of further strengthening the comparative aspect of our study. The six-weeks treatment period was selected according to our previous experimental protocol regarding OL's cardioprotection [26].

### 2.3 Animals and diet

In the present study, 161 male C57Bl6 mice and 30 male ApoE^−/−^ mice with C57Bl6 background, 10–12 weeks old, were included in 6 different series of experiments (Supplementary Fig. 2). The strain, age and sex selection, as well as the conduct of the surgical procedures were in compliance with “Practical guidelines for rigor and reproducibility in preclinical and clinical studies on cardioprotection”. Mice randomization was performed in all experiments, the surgical procedures and the analysis of the experimental data were performed in a blinded manner [43]. The required number of animals for statistical analysis was calculated by Gpower analysis (Supplementary Fig. 3). Mice were provided by the Centre of Clinical Experimental Surgery and Translational Research, Biomedical Research Foundation of the Academy of Athens, housed in the animal facility in standard conditions (21–24 °C and 40–80 % humidity) and maintained on a 12/12-h light/dark cycle. Fresh water and appropriate for mice standard chow (Normal Diet, ND) or Western Diet (WD) supplemented with 1.25 % cholesterol by Ssniff (#E15723-34, High Fat/High Cholesterol diet: 42 % Fat, 15 % Protein and 43 % Carbohydrates), were provided ad libitum according to ARRIVE guidelines [44,45].

All animal procedures were conducted in accordance with the Presidential Decree 56/2013 for the protection of animals used for scientific purposes, in harmonization with the European Directive 2010/63/EU for animal experiments. The experimental protocols were approved by the competent Veterinary Service of the Prefecture of Athens (License protocol numbers: 60858/23-01-20, 124351/15-02-22 and 349898/22-03-23).

### *2.4* *In vivo* experimental protocols

#### 2.4.1 1^st^series of experiments: Investigation of the cardioprotective effect of OL, HT, OC and OA in mice without MS

C57Bl6 mice were randomized into 5 groups: i. Vehicle (DMSO 5 %) (n = 4), ii. OL 20.6 mg/kg (n = 5), iii. HT 5.9 mg/kg (n = 5), iv. OC 11.6 mg/kg (n = 5) and v. OA 17.4 mg/kg (n = 4). Mice received the assigned treatment for 6 weeks daily by oral gavage [26,46] and then subjected to surgical induction of myocardial IRI by left anterior descending artery (LAD) ligation. The resulting infarct size was measured to reveal the potential cardioprotective properties of the compounds.

#### 2.4.2 2^nd^series of experiments: Investigation of OL, HT, OC and OA effects on myocardial IRI in the presence of MS

C57Bl6 mice were fed with WD for 14 weeks to induce MS [47]. On week 8, animals were randomized into 6 groups and received one of the isolated compounds or vehicle for the last 6 weeks daily by oral gavage as follows: i. Vehicle NS (n = 6), ii. OL 20.6 mg/kg (n = 7), iii. HT 5.9 mg/kg (n = 8), iv. Vehicle (DMSO 5 %) (n = 5), v. OC 11.6 mg/kg (n = 8) and vi. OA 17.4 mg/kg (n = 8). At baseline, on weeks 8 and 14, basic parameters associated with the MS, such as fasting blood glucose, total cholesterol, blood pressure, body and epidydimal adipose tissue weight were evaluated in all groups. Additionally, a group of healthy mice (n = 8) of the same age was used as controls, for comparative reasons. At the end of the 14^th^ week, mice with MS underwent myocardial IRI surgery for infarct size assessment.

#### 2.4.3 3^rd^series of experiments: Investigation of selected combinatorial treatments on myocardial IRI in mice with MS

According to the effects of each compound, three combinatorial treatments with expected additive cardioprotective effects were designed: Combo 1 (OL 20.6 mg/kg, HT 5.9 mg/kg and OA 17.4 mg/kg), Combo 2 (OL 20.6 mg/kg, HT 5.9 mg/kg and OC 11.6 mg/kg) and Combo 3 (OL 20.6 mg/kg, HT 5.9 mg/kg, OC 11.6 mg/kg and OA 17.4 mg/kg). To examine whether these combinations would confer to cardioprotection, MS was induced in mice receiving WD for 14 weeks, and they were randomized into the following 4 groups at the end of week 8: i. Vehicle (DMSO 5 %) (n = 6), ii. Combo 1 (n = 7), iii. Combo 2 (n = 8) and iv. Combo 3 (n = 6). Animals were treated with the assigned combination regimens for 6 weeks daily by oral gavage. At baseline, on weeks 8 and 14, MS induction and progression was evaluated in all groups, as previously described. At the end of week 14, mice were subjected to surgical IRI induction and infarct size was measured.

#### 2.4.4 4^th^series of experiments: cardioprotective mechanism of the most promising combinatorial treatment and its constituents in mice with MS

The experimental protocol described in 2.4.3 was repeated with the groups of mice receiving either Combo 2 (OL, HT, OC), which emerged as the most promising combinatorial treatment, since it presented favorable cardioprotective and antihyperglycemic potential, or each one of its constituents as follows (n = 8/group): i. Vehicle (DMSO 5 %), ii. OL 20.6 mg/kg, iii. HT 5.9 mg/kg, iv. OC 11.6 mg/kg and v. Combo 2 (OL, HT, OC). At the end of the 14th week, whole blood was collected for oxidized LDL (ox-LDL) and malondialdehyde (MDA) levels determination as described in the supplementary file, mice were subjected to IRI surgery and at the 2 h of reperfusion the heart was excised. The ischemic part was received and snap frozen for protein extraction and immunoblotting analysis.

#### 2.4.5 5^th^series of experiments: effect of the combinatorial treatment and its constituents on atherosclerosis development and progression *in vivo*

Male ApoE^−/−^ mice were fed with WD for 12 weeks to accelerate atherogenesis in large arteries [48]. At the 8^th^ week, mice were randomized into 5 groups (n = 6/group): i. Vehicle (DMSO 5 %), ii. OL 20.6 mg/kg, iii. HT 5.9 mg/kg, iv. OC 11.6 mg/kg and Combo 2 (OL, HT, OC). Animals received the respective treatment for 4 weeks daily by oral gavage [49]. At the end of 12 weeks, whole blood was collected for total cholesterol levels, ox-LDL and MDA determination as described in detail in the supplementary file. Mice were anesthetized with isoflurane (5 % in 1L/min oxygen for induction), were euthanatized via cervical dislocation, the descending thoracic aorta (including the distal aortic arch) was isolated for the measurement of the atherosclerotic plaque area via Oil Red O staining [50] and the assessment of ROS formation in aortic rings via the fluorescence of dihydroethidium (DHE) staining.

#### 2.4.6 6^th^series of experiments: multi-organ toxicity evaluation of the most promising combinatorial treatment

Combo 2 was evaluated in healthy animals for potential signs of multi-organ toxicity. C57Bl6 mice were randomized into 3 groups (n = 7/group): i. Vehicle (DMSO 5 %), ii. Combo 2 low dose (Combo 2 LD: 20.6 mg/kg, HT 5.9 mg/kg and OC 11.6 mg/kg) and iii. Combo 2 high dose (Combo 2 HD: OL 61.8 mg/kg, HT 17.7 mg/kg and OC 34.8 mg/kg). The animals received the assigned treatment for 4 weeks daily by oral gavage [51]. At the end of the 4-week treatment, whole blood was collected from the submandibular vein for the evaluation of blood, liver and kidney toxicity markers, aspartic aminotransferase (AST), alanine aminotransferase (ALT), lactic dehydrogenase (LDH), alkaline phosphatase (ALP), creatinine and urea. Mice were anesthetized with isoflurane (5 % in 1L/min oxygen for induction), were euthanatized via cervical dislocation and selected organs prone to injury (heart, liver, kidney, small intestine, pancreas, lung) were harvested for histological evaluation.

### 2.5 Murine model of IRI

Mice were anesthetized with a mixture of ketamine (100 mg/kg), xylazine (20 mg/kg) and atropine (0.6 mg/kg) administered intraperitoneally and remained under general anesthesia during the IRI procedures. The level of anesthesia was evaluated by loss of pedal reflex. Each animal was then placed on a heating pad, to maintain their temperature at 37 °C, and tracheotomy was performed to ensure mechanical ventilation, using a MiniVent ventilator for mice (Model 845, Harvard Apparatus), set at 200 μL tidal volume and 150 strokes/min respiration rate. After left sided thoracotomy and exposure of the heart, the pericardium was incised, and the LAD was ligated 3–4 mm distal to the origin of the artery under the left atrium using a 6–0 silk suture. Ligation was tightened for 30 min to block the regional coronary circulation through this artery and then released for 2 h to allow reperfusion of the ischemic area. Animals of the 1^st^, 2^nd^, 3^rd^ and 4^th^ series of experiments remained under general anesthesia until the end of the reperfusion and were subsequently euthanized by cervical dislocation and the heart was gently excised [28,43,52].

### 2.6 Infarct size measurement after IRI

Infarct size was evaluated by double staining of the heart with Evans blue and 2,3,5-Triphenyltetrazolium chloride (TTC). Promptly after 2 h of reperfusion, the myocardium was harvested, and the aorta was cannulated using an aortic metal cannula with luer taper (#73–2800 Harvard Apparatus) which was adopted to a screw needle cartridge. First, Krebs buffer (NaCl 118.5 mM, NaHCO_3_ 25.0 mM, KCl 47.0 mM, KH_2_PO_4_ 12.0 mM, MgSO_4_ 12.0 mM, Glucose 11.1 mM, CaCl_2_ 2.4 mM) was injected in the heart through the aorta, to remove the remaining blood and then the ligature was retightened and 2.5 % w/v Evans blue dye in Krebs buffer was inserted with a slow, steady flow to delineate the ischemic from the normally perfused area. Hearts were then sliced in 1 mm thick sections and incubated in 1 % w/v TTC in PBS solution (pH 7.4) for 10 min at 37 °C. Therefore, viable areas of the heart tissue were stained red, while necrotic areas remained unstained (white) [43]. Photos of both sides of each slice were obtained (Olympus EP50 digital camera on Olympus SZ61 stereoscope) to determine the area at risk and the infarct size by planimetry using ImageJ software and calculate Infarct/Risk and Risk/All ratios % [52].

### 2.7 Arterial blood pressure measurement

Non-invasive blood pressure measurement was performed on awake mice using CODA Monitor tail-cuff system (Kent Scientific Co, Torrington, CT USA). The mice were placed for 10 min inside a heated chamber (34 °C) and then positioned in a restrainer over a heating pad. They were allowed a 5-min acclimation period following cuff positioning and 20 consecutive blood pressure determination cycles were conducted. All measurements were recorded on the CODA software [47]. In case of signs of discomfort, the animal was returned to the heated chamber and re-examined 1 h later. The researchers were blinded to the study.

### 2.8 Echocardiography

Echocardiography was performed in the 2^nd^ and 3^rd^ experimental series, on mice receiving WD and treated either with the isolated compounds or the selected mixtures at three timepoints: baseline, 8^th^ week (before treatments) and 14^th^ week (after treatments). A Vevo 2100 ultrasound system (VisualSonics, Toronto, Canada) equipped with a MS400 transducer was used by an experienced sonographer in a blinded manner. Mice were anesthetized with isoflurane (5 % in 1L/min oxygen for induction, and 0.5–1% for maintenance of anesthesia). The mouse limbs were taped to four electrocardiogram electrodes, which were embedded in a heated platform for the measurement of heart rate, electrocardiogram (ECG) and respiratory rate. 2D targeted M-mode imaging was obtained to measure left ventricular (LV) end-diastolic diameter (LVEDD), LV end-systolic diameter (LVESD), and LV posterior wall thickness at systole (PWTs) and diastole (PWTd). End diastole was determined at the maximal LV diastolic dimension, and end systole was evaluated at the peak of posterior wall motion. The LV radius to PWT ratio (r/h), the percentage of LV fractional shortening (FS %) and ejection fraction (EF %) were also calculated, as previously described. Three beats were averaged for each measurement [53].

### 2.9 Histology

#### 2.9.1 Paraffin sections

Fresh tissue was fixed in 10 % buffered formalin and then embedded in paraffin. Sections of each sample with thickness 5 μm were obtained. Tissue specimens of the toxicity study were routinely stained with hematoxylin and eosin (H&E) [53]. Specimens were observed and photographed on bright-field LEICA DMLS2 microscope and assessed by a certified blinded pathologist for signs of inflammation or tissue injury.

#### 2.9.2 Cryosections

Unfixed tissue sample was immersed in OCT embedding medium by quick freezing in liquid nitrogen. Using a cryostat (Leica microsystems GmbH, Wetzlar, Germany) consecutive cryosections 8 μm thick were obtained and mounted onto electrostatically adherent slides (Fisherbrand™ Superfrost™ Plus). The specimens were then stained with Oil Red O to evaluate the atherosclerotic plaque area and DHE for reactive oxygen species (ROS) determination [50,54]. Relative densitometry of the Oil Red O positive area was performed using ImageJ software. Atherosclerosis burden was quantified as Oil Red O-stained area as a percentage of total aortic area [55]. Cryosections 10 μm thick were cut and treated with DHE in PBS (5 × 10^−6^ M concentration; 200-μL volume per section) or with PBS (200 μL volume; vehicle/time control). All sections were incubated at room temperature in a light-protected, humidified chamber for 30 min, and rinsed twice with PBS to remove unoxidized DHE. After washing, specimens were treated with VECTASHIELD antifade mounting medium with 49,6‐diamidino‐2‐phenylindole (DAPI) for DNA staining (Vektor, cat. H‐1200) and visualized in Zeiss PALM MicroBeam IV Laser-capture Microdissection System using the DAPI filter for the nuclei visualization, the fluorescein isothiocyanate (FITC) filter for DHE visualization and the tetramethylrhodamine (TRITC) filter for visualization of the auto-fluorescence of elastin. The settings for imaging included a 2464 × 2056 pixel resolution, exposure time/gain 350 ms/1 for DAPI, 1000 ms/for FITC and 500 ms/2 for TRITC), and objective: × 40, and were identical for all sections. At least three fields of view from each vascular ring were imaged such that no regional overlap occurred. The mean gray value for each acquisition was determined via the ImageJ the relative fluorescence intensity of DHE/DAPI was used for statistical analysis [54].

### 2.10 Neutrophil isolation and Neutrophil Extracellular Traps (NETs) formation

Neutrophils were isolated from whole blood of healthy volunteers, as previously described [56]. Neutrophil suspension, in serum free Roswell Park Memorial Institute (RPMI)-1640 medium, was added in a 24-well plate (200.000 neutrophils/well), while a glass coverslip was already placed in each well, in a final volume of 500 μl culture medium RPMI-1640, supplemented with 2 % v/v heat-inactivated human serum. The plate was incubated for 1 h at 37 °C, 5 % CO_2_. The neutrophils were incubated with 25 μg/ml and 50 μg/ml of the isolated compounds, their combination or their vehicle (DMSO) for 10 min and then activated with Phorbol 12-myristate 13-acetate (PMA) for 3.5 h at 37 °C, 5 % CO_2_. The highest concentration of 50 μg/ml was calculated based on the expected circulating levels of HT. The dose of 5.9 mg/kg with a mean weight of 35g in mice with MS, blood volume of 2.8 mL per mouse and the previously reported 75 % bioavailability of hydroxytyrosol in aqueous solutions [18], results in an expected 55.3 μg/mL concentration in blood and for this reason, we tested the higher dose of 50 μg/mL for *in vitro* experiments and half of this dose. For the combination treatment we used 16.66 μg/ml from each compound to achieve a final concentration of 50 μg/ml and 8.33 μg/ml for the 25 μg/ml, respectively. We performed the same assay, without activating the neutrophils with PMA, to investigate if the compounds would promote Neutrophil Extracellular Traps (NETs) formation in absence of PMA stimulation. Neutrophils were then fixed and incubated with the primary anti-Myeloperoxidase (MPO) [Santa Cruz Biotechnology (266-6K1): sc-52707] and the secondary antibody [goat anti-rabbit IgG (H + L), conjugated with Alexa Fluor® 488, Invitrogen A11034] for 1 h in the dark, respectively. After cell staining with DAPI for 10 min in the dark, the coverslips were placed on slides for the immunofluorescence assay [57]. Neutrophils were observed under a microscope (Olympus Fluorescence Microscope, BX41, magnification 40Χ) and the percentage of NETosis was determined by counting the number of neutrophils that formed NETs in five representative fields of each condition. The results were extracted from three independent experiments.

### 2.11 Clinical study design

A prospective, randomized cross-over, double-blind, placebo-controlled clinical study was designed to investigate whether olive oil supplemented with OL, HT and OC based on Combo 2 synthesis would benefit patients at high cardiovascular risk. To this end, 15 patients with CCAS were recruited after providing a written consent and were randomized into 2 groups (Supplementary Fig. 4). Each participant underwent echocardiographic evaluation and blood collection at baseline and then received either 4 capsules of the supplement (OL, HT, OC-enriched olive oil) or 4 capsules of placebo daily for one month. After the first month, each patient treated with placebo was assigned the supplement daily for one month, while patients who received the supplement were treated with the placebo daily for one month. Placebo capsules contained olive oil and had the same organoleptic properties (label, taste, shape and colour) with the capsules enriched with OL, HT and OC as we have previously used [58]. At the end of each treatment, all patients repeated the full echocardiographic evaluation and blood collection.

The supplement's effects on endothelial glycocalyx thickness, endothelial function, arterial stiffness, coronary function and left ventricular function in patients with CCAS, were defined as primary endpoints of the study, which was approved by the Attikon University Hospital scientific ethics committee (Approval number: 507/06-07-2023) and conducted according to the Declaration of Helsinki. Written informed consent was provided by all participants. The detailed methodology for the assessment of perfused boundary region (PBR), flow-mediated dilation (FMD) and Pulse Wave Velocity (PWV) indicative of endothelial glycocalyx thickness, endothelial function and arterial stiffness respectively as well as for the coronary and left ventricular function in patients is described in the supplementary file. Registration of the clinical trial on website: https://clinicaltrials.gov/, NCT04520126, 17/08/2020.

The inclusion criteria for the recruitment were patients 18–70 years old, diagnosed with CCAS according to the current guidelines (ESC 2019) [59] or patients who experienced an acute coronary syndrome and have undergone percutaneous coronary intervention (PCI) or coronary artery bypass grafting, on the condition that 2 or more months have passed since the event. Exclusion criteria included age >70 years old or <18 years old, diabetes mellitus, uncontrolled hypertension, hepatic, thyroid and inflammatory diseases, malignancies or chronic renal failure (creatinine >2 mg/dL). Furthermore, it was necessary that patients had not joined a weight-loss program, participated in any exercise training program, or had any nutritional intervention for the last two months and/or concomitant multivitamin supplementation with antioxidant effects. Demographic and clinical characteristics (medical record, vital signs and current medications) were recorded and are presented in Supplementary Table 10.

Dose regimen was selected according to our *in vivo* experimental findings which determined the dosage ratio of each compound (OL:HT:OC 20.6:5.9:11.6 mg/kg of body weight) and by taking into consideration the encouraging results of our previous clinical trial, which included HT-enriched olive oil [60]. Researchers and patients were both blinded during the study. The supplement and placebo were formulated in soft capsules and kindly provided by Unipharma S.A.

### 2.12 Statistical analysis

#### *2.12.1* *In vitro* and *in vivo* results

All results were statistically analyzed using GraphPad Prism 8 software (Graph Pad Software, Inc.) and data are plotted as means ± standard error of means (mean ± SEM). Each data point represents a biological replicate. For comparison between 2 groups unpaired, two-tailed Student's t-test was implemented. For comparisons among three or more groups by one parameter, one way analysis of variance (ANOVA) was performed. Multiple comparisons among all means were conducted with Tukey's post hoc test. In the *in vivo* toxicity study, Dunnett's post hoc test was performed to compare each mean with the mean of the vehicle-treated group. For parameters not following normal distribution within groups, according to the Kolmogorov-Smirnov normality test, the non-parametric Kruskal-Wallis test was employed with Dunn's multiple comparisons post hoc test. Outlying values were identified by ROUT (Q = 1 %) test and excluded from analysis. In graphs including two parameters (treatment and timepoint during WD administration) Two-way ANOVA was performed to evaluate the effect of each parameter in the studied variable means. To examine the effect of WD at the three timepoints (Baseline, 8 weeks, 14 weeks) within each group, Tukey's post hoc test was used for multiple comparisons. Tukey's post hoc test was also employed to examine the effect of every treatment in each timepoint, when 3 or more groups were compared, while Sidak post hoc test was used when two groups were compared (DMSO 5 % vs NS vehicle groups). P values < 0.05 are considered statistically significant and are reported accordingly.

#### 2.12.2 Clinical study results

The results from the clinical trial were analyzed using the SPSS v.22 software. Data are presented as mean ± standard deviation (SD) or median with interquartile range (25^th^-75^th^ percentile), if not normally distributed. The Shapiro-Wilk test was utilized to assess the normal distribution of the data, while the Levene test was employed to evaluate data homoscedasticity. Non-parametric variables were converted to ranks for analysis. All analyses employed two-tailed tests with a significance level set at p < 0.05. A p value below 0.05 was considered statistically significant. Statistical differences for each measured variable were evaluated using independent samples t-tests or paired t-tests for normally distributed continuous variables. For continuous variables with non-parametric distribution, the Mann-Whitney test or Wilcoxon test was applied. Categorical variables were assessed using a chi-squared or Fisher's exact test, as appropriate. Repeated measures ANOVA was conducted for the baseline, one month after placebo treatment, and one month after active product treatment, treating intervention as a within-subject factor. F and p values for treatment comparisons were calculated. If the sphericity assumption, assessed by Mauchly's test, was violated, the Greenhouse-Geisser correction was applied. Paired t-tests were utilized to compare the percent changes in examined biomarkers after treatment (supplement vs placebo).

## 3 Results

### 3.1 Chronic pretreatment with OL, OC and OA at nutritional dose reduces myocardial infarct size in healthy and MS-burdened mice

Our first goal was to evaluate whether the nutraceutical doses of olive constituents protect the heart against IRI in the absence of MS. Hence, mice received either the isolated compounds OL, HT, OC, OA or DMSO 5 % for 6 weeks before surgical IRI induction. Our results demonstrate that OL, OC and OA, but not HT, significantly reduced infarct size compared to the Veh (DMSO 5 %) group (Supplementary Fig. 5A). Area at risk was similar among groups, indicating that the surgical procedure provoked an ischemic insult of similar extent in all groups (Supplementary Fig. 5B).

We then aimed to examine whether the bioactive olive constituents retain their infarct size limiting properties in the presence of MS, since comorbidities, such as hypercholesterolemia, blunt the cardioprotective effect of several cardioprotective modalities [61]. At the 8^th^ week, successful MS induction by WD administration was validated by the coexistence of disturbed glucose homeostasis, as indicated by increased fasting glucose levels (Supplementary Fig. 6A), glucose intolerance (Supplementary Figs. 6B and C), hypercholesterolemia (Supplementary Fig. 6D) and obesity implied by the higher epididymal adipose tissue relative weight (Supplementary Fig. 6E) and the elevated body weight (Supplementary Fig. 6F). At the 14^th^ week, these disturbances were either similar or exacerbated, ensuring the MS establishment. Blood pressure was not altered throughout the establishment of the MS (Supplementary Fig. 6G). Moreover, echocardiographic analysis showed that FS% and EF% decreased at 8 and 14 weeks in the Veh (DMSO 5 %) but within normal limits [62] and without any significant impact in overall cardiac function (Supplementary Table 1).

**Fig. 1:**
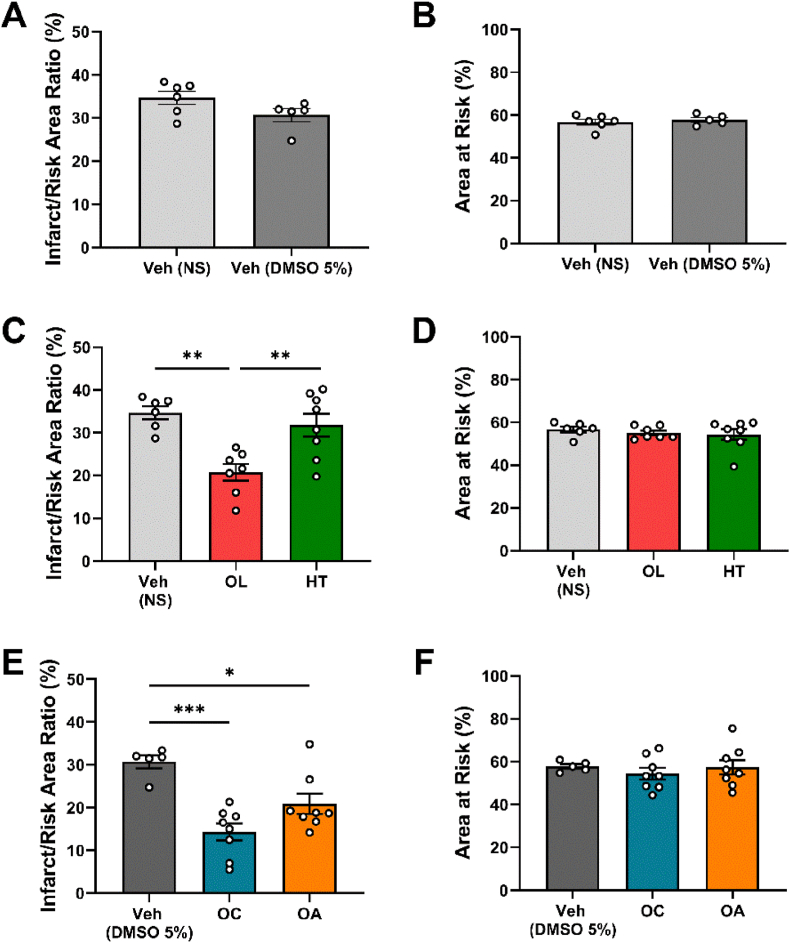
**Oleuropein, oleocanthal and oleanolic acid reduce infarct size in MS-burdened mice.***Graphs of****A.****Infarct/Risk Area Ratio % and****B.****Area at risk % of NS- and DMSO 5%-treated mice.****C.****Infarct/Risk Area Ratio % and****D.****Area at risk % of NS-, OL- and HT-treated mice.****E.****Infarct/Risk Area Ratio % and****F.***Area at risk % of DMSO 5%-, OC- and OA-treated mice. A, B Unpaired t-test. C, D, E, F One-way ANOVA, Tukey post hoc test. *p < 0.05, **p < 0.01, ***p < 0.005. All values are presented as Mean ± SEM (n = 5-8). DMSO: Dimethyl sulfoxide; HT: Hydroxytyrosol; NS: Normal saline; OA: Oleanolic Acid; OC: Oleocanthal; OL: Oleuropein.

Consequently, at the end of week 14, the mice were subjected to 30 min of myocardial ischemia, followed by 2h reperfusion and the infarct size was measured. The vehicle-treated groups, namely the NS and DMSO 5%-treated mice, exhibited similar results in the calculated parameters, indicating that DMSO 5 % does not affect the myocardial infarct size (Fig. 1A and B). Pretreatment with isolated OL, OC and OA significantly reduced the infarct size compared to the respective vehicle group. Nevertheless, treatment with HT did not affect the infarct size (Fig. 1C and E), in compliance with the wild-type mice without MS (Supplementary Fig. 5A). No differences were observed in the area at risk among the groups (Fig. 1D and F).

### 3.2 OL mitigated hyperglycemia and increased insulin levels in plasma

**Fig. 2:**
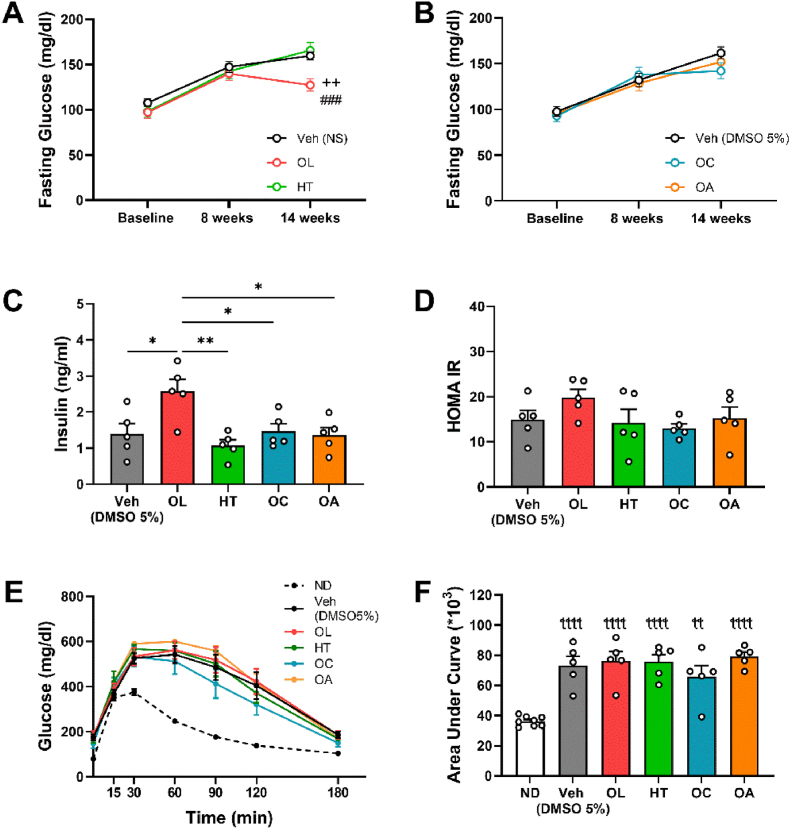
**Oleuropein attenuates hyperglycemia and increases insulin levels in plasma.***Graphs of***A.***Fasting blood glucose of NS-, OL- and HT-treated mice.***B.***Fasting blood glucose of DMSO 5%-, OC- and OA-treated mice.***C.***Fasting insulin levels in plasma at the 14*^*th*^*week.***D.***Calculated HOMAIR index.***E.***Glucose curves for all treated groups and the ND group.***F.***Calculated Area Under Curve for each glucose curve, including the ND group. A, B Two-way ANOVA, Tukey post hoc test. C, D, F One-way ANOVA, Tukey post hoc test.*^*++*^*p < 0.01 vs Veh (DMSO 5 %),*^*###*^p < 0.001 vs HT, *p < 0.05, **p < 0.01,^*ꞎꞎ*^*p<0.01 vs ND,*^*ꞎꞎꞎꞎ*^*p<0.0001 vs ND. All values are presented as Mean ± SEM (n = 5-13). DMSO: Dimethyl sulfoxide; HT: Hydroxytyrosol; ND: Normal Diet; OA: Oleanolic Acid; OC: Oleocanthal; OL: Oleuropein.*

Treatment with OL resulted in significantly decreased fasting glucose levels at 14 weeks compared to the Vehicle (NS) group, whilst groups treated with HT, OC or OA presented no differences compared to the respective vehicle-treated groups (Fig. 2A and B). At the same time, fasting insulin levels in plasma were determined and found significantly increased only in OL group, compared to every other group (Fig. 2C). The marker Homeostatic Model Assessment for Insulin Resistance (HOMAIR) [63] did not reveal any beneficial effects on insulin resistance by any of the treatments (Fig. 2D). Also, the Glucose Tolerance Test (GTT) resulted in similar glucose curves (Fig. 2E) with no significant difference in Area Under Curve (AUC) among the vehicle and treatment groups, but significantly different from the non-diabetic group (Fig. 2F). In total, OL mitigated hyperglycemia and increased plasma insulin levels, without affecting the glucose intolerance and insulin resistance.

### 3.3 OA limited hypercholesterolemia, while OL, HT and OC did not affect the circulating lipids. None of the bioactive compounds mitigated obesity

**Fig. 3:**
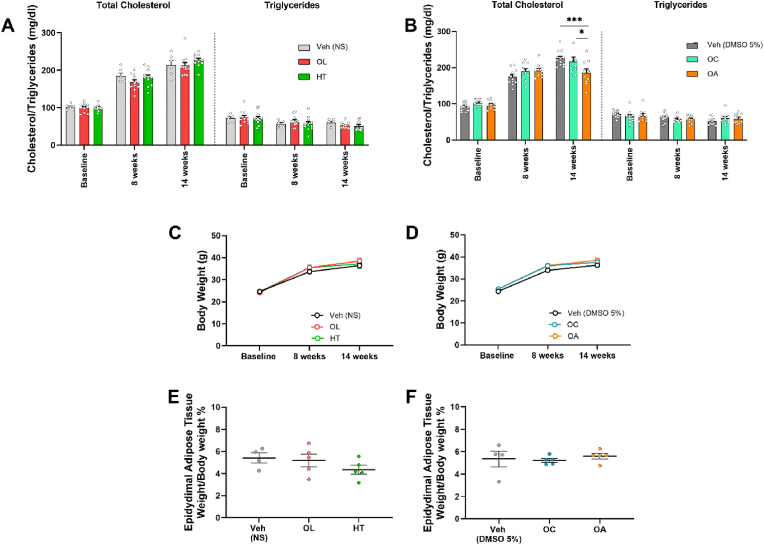
**Oleanolic*****a*****cid reduces total cholesterol but none of the treatments mitigates obesity.***Graphs of***A.***Total cholesterol and triglycerides levels of NS-, OL- and HT-treated mice (mg/dl).***B.***Total cholesterol and triglycerides levels of DMSO 5%-, OC- and OA-treated mice (mg/dl).***C.***Body weight of mice treated with NS, OL, HT.***D.***Body weight of mice treated with DMSO 5 %, OC, OA.***E.***Relative weight of epidydimal adipose tissue in mice treated with NS, OL, HT.***F.**Relative weight of epidydimal adipose tissue in mice treated with DMSO 5 %, OC, OA. A, B, C, D Two-way ANOVA, Tukey post hoc test. E, F One-way ANOVA, Tukey post hoc test. *p < 0.05, ***p < 0.001. All values are presented as Mean ± SEM (n = 5-14). HT: Hydroxytyrosol; ND: Normal Diet; OA: Oleanolic Acid; OC: Oleocanthal; OL: Oleuropein.

The assessment of plasma cholesterol and triglycerides revealed that treatment with OA significantly lowered total cholesterol compared to the Vehicle at 14 weeks, whilst triglycerides levels did not differ among the groups (Fig. 3A and B). Furthermore, 6 weeks of treatment with the isolated olive constituents (weeks 8–14) did not significantly affect the constantly increasing body weight (Fig. 3C and D), and adipose tissue relative weight (Fig. 3E and F). In total, none of the bioactive compounds could reverse the induction and progression of obesity, but OA attenuated hypercholesterolemia. No significant alterations were observed in blood pressure (Supplementary Table 2) and cardiac function (Supplementary Table 3).

### 3.4 All selected combinatorial treatments reduced myocardial infarct size in the presence of MS

**Fig. 4:**
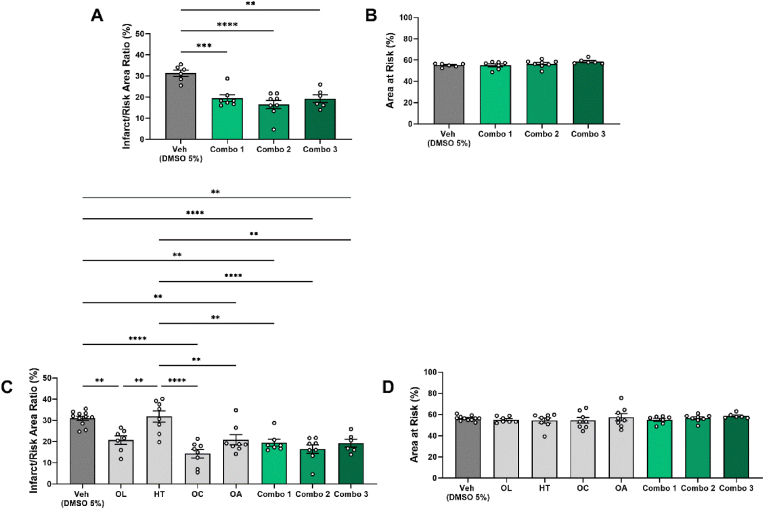
**Combinatorial treatments 1, 2 and 3 significantly reduce the infarct size in similar extent to the cardioprotective oleuropein, oleocanthal and oleanolic acid.***Graphs of****A.****Infarct/Risk Area Ratio % and****B.****Area at Risk % of mice treated with combinatorial treatments or DMSO 5 %.****C.****Infarct/Risk Area Ratio % and****D.***Area at Risk % of all mice treated with the isolated compounds or combinations. A, B, C, D One-way ANOVA, Tukey post hoc test. **p < 0.01, ***p < 0.001, ****p < 0.0001. All values are presented as Mean ± SEM (n = 6-11) Combo: Combinatorial therapy; DMSO: Dimethyl sulfoxide; HT: Hydroxytyrosol; ND: Normal Diet; OA: Oleanolic Acid; OC: Oleocanthal; OL: Oleuropein.

Since **i.** HT has an EFSA claim for its prevention against LDL oxidation, and we have shown that **ii.** OL decreases infarct size and improves glucose homeostasis, **iii.** OC exerts infarct-sparing effects and **iv.** OA mitigates infarct size and decreases total cholesterol, we designed three combination therapies, namely Combo 1 (OL, HT, OA), Combo 2 (OL, HT, OC) and Combo 3 (OL, HT, OC, OA) (Supplementary Table 4). Significantly reduced infarct size was observed after the administration of all three of the combinatorial treatments compared to the vehicle group. No statistically significant differences were observed among the treated groups, suggesting their equal cardioprotective potential (Fig. 4A). Area at risk was similar among all groups (Fig. 4B).

Subsequently, we aimed to clarify the existence or absence of additive cardioprotective effects. Therefore, we retrospectively incorporated the results concerning the infarct size, after administration of the isolated compounds and statistically compared with the combinatorial treatments and the vehicle group. All the treatments that exhibited cardioprotection in our study (OL, OC, OA, Combo 1, Combo 2 and Combo 3) presented significantly reduced infarctions, compared to both the non-cardioprotective HT and their respective vehicle groups. However, comparison among treatments did not reveal differences in the cardioprotective potency, indicating that the combinatorial treatments do not exhibit additive and/or synergistic impact in terms of myocardial infarct size reduction at the selected nutritional doses (Fig. 4C and D).

### 3.5 Effect of combinatorial treatments on MS and cardiac function

**Fig. 5:**
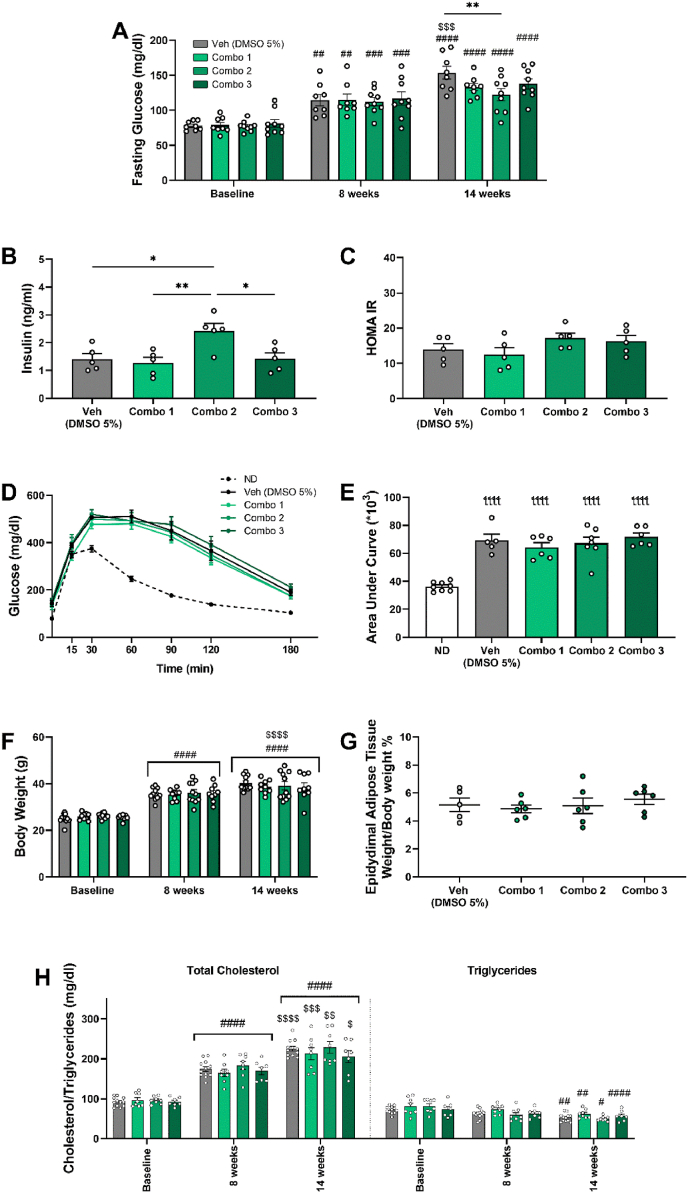
**Combo 2 (OL, HT, OC) reduces fasting glucose levels and increases insulin in plasma, with no significant effects on hypercholesterolemia and obesity.***Graphs of****A.****Fasting blood glucose of DMSO 5%-, Combo 1-, Combo 2- and Combo 3- treated mice.****B.****Fasting insulin level in plasma at the 14*^*th*^*week.****C.****Calculated HOMAIR index.****D.****Glucose curves.****E.****Calculated Area Under Curve for each glucose curve.****F.****Body weight of DMSO 5%-, Combo 1-, Combo 2- and Combo 3- treated mice.****G.****Relative adipose tissue weight.****H.****Total cholesterol and Triglycerides in plasma. A, F, H Two-way ANOVA, Tukey post hoc test. B, C, E, G One-way ANOVA, Tukey post hoc test.*^*##*^*p < 0.01,*^*####*^*p < 0.0001 vs Baseline,*^*$*^*p < 0.05,*^*$$*^*p < 0.01,*^*$$$*^*p < 0.001,*^*$$$$*^*p < 0.0001 vs 8 weeks,*^*ꞎꞎꞎꞎ*^p<0.0001 vs ND, *p < 0.05, **p < 0.01. All values are presented as Mean ± SEM (n = 5-11). DMSO: dimethyl sulfoxide; Combo 1: OL, HT, OA; Combo2: OL, HT, OC; Combo 3: OL, HT, OC, OA.

Since the three combinatorial treatments exhibited similar cardioprotective properties against IRI, we further examined their impact on MS progression and cardiac function, to pinpoint additional cardiovascular benefits. The elevated fasting glucose was reduced by the pretreatment with Combo 2 at 14 weeks compared to the Veh (DMSO 5 %) group (Fig. 5A). Combo 2 was also the only treatment that increased fasting insulin levels compared to the other groups (Fig. 5B), with no significant effects being observed in the insulin resistance marker HOMAIR (Fig. 5C). Quantification of the AUC after GTT showed significantly higher values than the normal diet-treated mice in all four groups, indicating that glucose intolerance caused by WD persisted after chronic treatment with the combination therapies (Fig. 5D and E). Body weight (Fig. 5F), adipose tissue mass (Fig. 5G), total cholesterol and triglycerides (Fig. 5H) did not differ among the groups.

As Combo 1 and Combo 3 did not show the anticipated antihyperglycemic and anti-hypercholesterolemic effect, we also ensured the stability, chemical integrity, and possible interactions between the compounds of the mixture. ^1^H NMR analysis (Supplementary Fig. 7 and Supplementary Tables 5 and 6) showed no differences in the spectroscopic characteristics, namely protons chemical shift, peaks’ integration and multiplicity of OL and OA when analyzed as single compounds or in mixture, in the same concentration. This finding indicates that the chemical structure is preserved in the treatment solution and no chemical interactions are observed.

Echocardiographic evaluation of Combo 1 group revealed a significant reduction in FS% and EF% compared to the Veh (DMSO 5 %) but all measured parameters remain within normal range [62]. Combo 2 and 3 groups presented with no alterations in LV function in comparison to the vehicle-treated group. Animals in Combo 3 group present with a thickening on the posterior ventricle wall only in systole. Overall, the combination treatments do not have a negative impact on myocardial function since there are no pathologic signs in the rest of the studied parameters (Supplementary Table 7).

### 3.6 Cardioprotective and antioxidant mechanism of Combo 2 and its constituents

The combinatorial treatment manifesting the most favorable beneficial effects on IRI and MS parameters, namely Combo 2, was then further investigated concerning the underlying cardioprotective mechanism of action in comparison to all the isolated compounds separately.

**Fig. 6:**
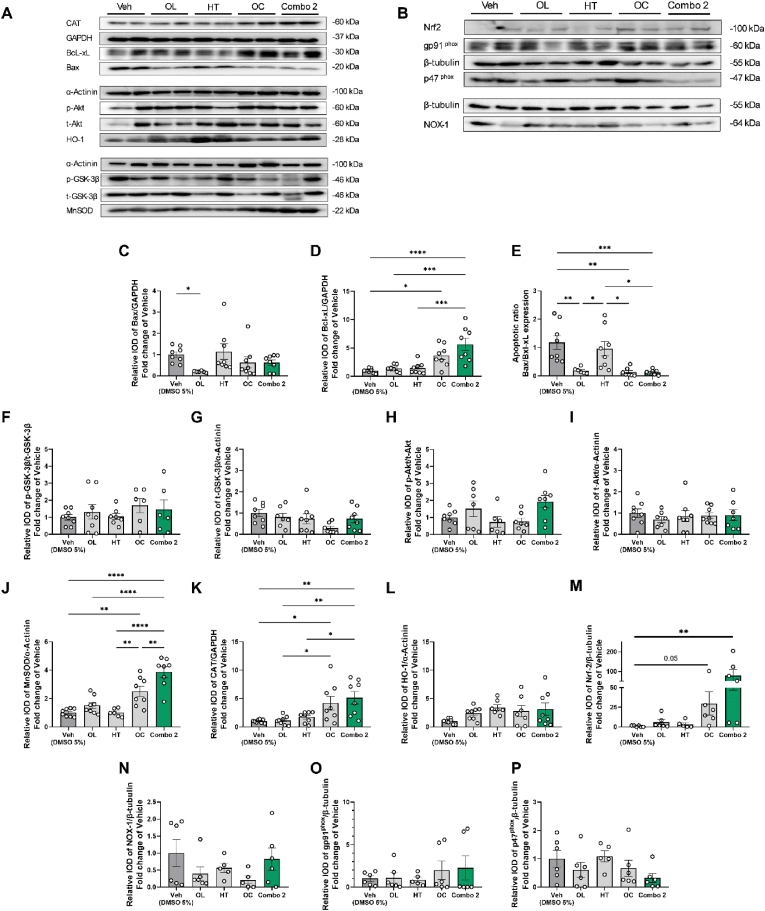
**Oleuropein, Oleocanthal and Combo 2 reduce the apoptotic ratio of Bax/BcL-xL, while Oleocanthal and Combo 2 additionally upregulate Nrf2 levels and the antioxidant enzymes MnSOD and CAT*****.*****A.***Representative blots at 2 h of reperfusion of CAT, GAPDH, Bax, BcL-xL, α-Actinin, p-Akt (S473), t-Akt, HO-1, p-GSK-3β (S9), t-GSK-3β and MnSOD.****B.****Representative blots at 2 h of reperfusion of Nrf2, gp91*^*phox*^*, p47*^*phox*^*, NOX-1 and β-tubulin. Relative densitometric graphs after normalization to total protein of***C.***Bax/GAPDH.***D.***BcL-xL/GAPDH.***E.***Bax/BcL-xL.***F.***p-GSK-3β (S9)/t-GSK-3β.***G.***t-GSK-3β/α-Actinin.***H.***p-Akt (S473)/t-Akt.****I.****t-Akt/α-Actinin.***J.***MnSOD/a-Actinin.***K.***CAT/GAPDH.***L.***HO-1/α-Actinin.***M.***Nrf2/β-tubulin,****N.****NOX-1/β-tubulin,****O.****gp91*^*phox*^*/β-tubulin,****P.****p47*^*phox*^/β-tubulin.C, M, N, O, P Kruskal-Wallis, Dunn's post hoc test. D, E, F, G, H, I, J, K, L, One-way ANOVA, Tukey post hoc test. *p < 0.05, **p < 0.01, ***p < 0.001, ****p < 0.0001. All values are presented as Mean ± SEM (n = 6-8). Akt: Protein Kinase B; Bax: BCL2 Associated X; Bcl-xL: B-cell lymphoma-extra-large; CAT: Catalase; Combo 2: Combinatorial treatment with OL, HT, OC; DMSO: Dimethyl sulfoxide; GAPDH: Glyceraldehyde-3-phosphate dehydrogenase; HO-1: Heme oxygenase 1; HT: Hydroxytyrosol; MnSOD: Manganese-superoxide dismutase; ND: Normal Diet; NOX -1: NADPH oxidase 1, Nrf2: nuclear factor erythroid 2–related factor 2;OC: Oleocanthal; OL: Oleuropein.

Firstly, we focused on apoptosis, and we observed a significant reduction on the expression of the apoptotic mediator Bax in the ischemic part of the heart, after chronic pretreatment with OL compared to the Veh (DMSO 5 %) group at 2 h of reperfusion (Fig. 6A and C). Meanwhile, the expression of the antiapoptotic mediator B-cell lymphoma-extra large (BcL-xL) was significantly elevated by OC and Combo 2 pretreatment compared to the Veh (DMSO 5 %) group (Fig. 6A and D). To gain improved insight on apoptotic/antiapoptotic balance in the reperfused myocardium, we also assessed the apoptotic ratio as Bax/BcL-xL [64]. Our results indicated a significantly reduced Bax/BcL-xL ratio after OL, OC and Combo 2 treatments compared to both the Veh (DMSO 5 %) and the HT group (Fig. 6A and E).

We subsequently investigated whether the antiapoptotic effect could be attributed to the Reperfusion Injury Salvage Kinase (RISK) pathway activation [65], and, therefore, we evaluated and compared the total expression and phosphorylation levels of protein kinase B (Akt) and glycogen synthase kinase 3 beta (GSK-3β) proteins at 2 h of reperfusion [65]. Treatment with Combo 2 or its isolated constituents induced no alterations in Akt and GSK-3β phosphorylation and expression, implying that cardioprotection at the selected timepoint is not due to the RISK pathway activation (Fig. 6A and 6F-I).

Next, based on numerous reports regarding the putative potential of olive constituents to upregulate endogenous antioxidant defense mechanisms [66], we hypothesized that the cardioprotective effects may be linked to their effect on oxidative stress. Thus, we assessed the levels of three antioxidant enzymes, namely manganese-superoxide dismutase (MnSOD), heme-oxygenase-1 (HO-1) and catalase (CAT). Pretreatment with OC as well as Combo 2, significantly increased the expression of MnSOD in comparison to the Veh (DMSO 5 %) group in the ischemic myocardium. Notably, the upregulation of MnSOD by Combo 2 was significantly higher than OC, highlighting a more potent effect exhibited by the combinatorial treatment (Fig. 6A and J). 10.13039/100014337Furthermore, 10.13039/100004749CAT was elevated in animals treated with OC and Combo 2, supporting the hypothesis of endogenous antioxidant defense enhancement, as part of the mechanism of action of OC and Combo 2 (Fig. 6A and K) None of the treatments induced a statistically significant alteration in the HO-1 levels (Fig. 6A and L). Nuclear factor erythroid 2-related factor 2 (Nrf2) is a transcription factor which orchestrates the cellular defense against oxidative insults through the expression of antioxidant enzymes including MnSOD and CAT [67,68] and has been associated with protection against ischemic injury [69,70]. Our results demonstrate that at 2 h of reperfusion OC and Combo 2 pretreatment increased Nrf2 protein levels (p = 0.05 for oleocanthal and p < 0.01 for Combo 2 in comparison to the vehicle treated group, Fig. 6B and M), in line with the upregulation of MnSOD and CAT, suggesting that is linked to their infarct size limiting effects.

NADPH oxidases (NOX) is a family of enzymes responsible for ROS production within mitochondria. Among the NOX family members, NOX-1, NOX-2 (also known as gp91^phox^) and NOX4 and their regulatory subunit p47^phox^ are important ROS contributors in CVDs [71] and have been proposed as targets of olive-oil derived bioactive compounds [72]. We found that OL, HT, OC and Combo 2 did not alter the expression of NOX-1, gp91^phox^ and p47^phox^ in the ischemic myocardium (Fig. 6B, N, O and P), suggesting that the cardioprotective mechanism is not linked to ROS production by the NOX system. Nonetheless, nitrotyrosine (NT) formation, as an index of tyrosine induced modification by peroxynitrite and nitro-oxidative stress [73] was alleviated by HT, OC and Combo 2 (Supplementary Figs. 8A and 8B) and had significantly lower levels than OL alone, further confirming the enhanced antioxidant effect of Combo 2 in comparison to the isolated bioactive constituents. Subsequently, we hypothesized that olive-derived compounds could alleviate systemic oxidative stress burden and we determined MDA levels as a marker of lipid peroxidation in the circulation [46] and the levels of ox-LDL. HT and Combo 2 significantly reduced MDA levels (Supplementary Fig. 8C) while HT, OC and Combo 2 significantly attenuated ox-LDL levels. In support of the effect on LDL oxidation *in vivo*, pretreatment with 10.13039/100014246HT or OC or Combo 2 led to decreased LDL-oxidation products *in vitro* (Supplementary Fig. 8E).

### 3.7 Pretreatment with HT, OC or Combo 2 inhibited NETs formation *in vitro* and intercepted atherosclerotic plaque development *in vivo*

**Fig. 7:**
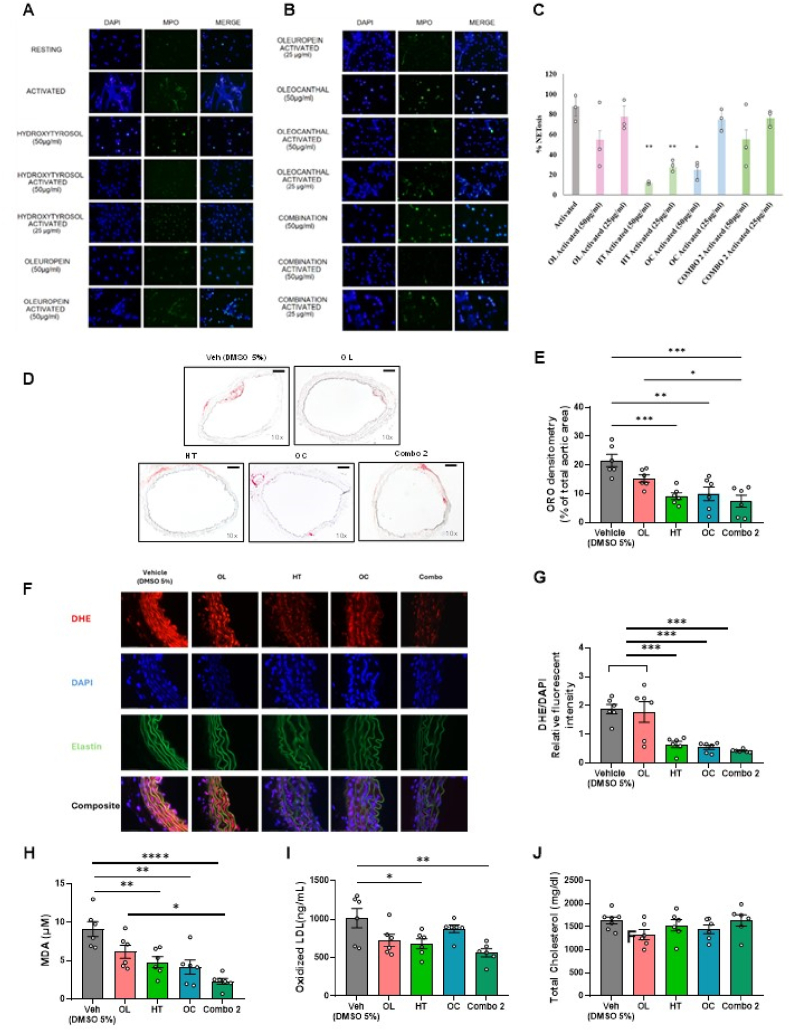
**Hydroxytyrosol and Oleocanthal limit NETosis *in vitro*, while Hydroxytyrosol, Oleocanthal and Combo 2 significantly reduce atherosclerotic lesion extent and oxidative stress *in vivo*.***Representative images of****A.****and****B.****the effect of the isolated compounds and their combination on neutrophil MPO release, with or without PMA activation, under fluorescent microscope and****C****the relative quantification of % netosis for each treatment.****D.****ApoE*^−/−^*aortic sections stained with Oil Red O under brightfield microscope (scale is* 150 μm*) and****E****. Oil Red O (ORO) densitometry (% of total aortic area) in aortic rings of ApoE*^−/−^*mice after 4-week treatment with OL, HT, OC or Combo 2.****F.****Representative images of aortic segments stained with DHE and DAPI to identify reactive oxygen species formation (scale is 75μΜ) and****E.****bar plots with relative fluorescent intensity quantification of DHE/DAPI for all experimental groups after 4-week treatment with OL, HT, OC or Combo 2. Bar plots of****H****. MDA and****I.****ox-LDL and****J****. total plasma cholesterol in the circulation of ApoE*^−/−^mice after the 4-week pretreatment. E, G, H, I, J One-way ANOVA, Tukey post hoc test. *p < 0.05, **p < 0.01, ***p < 0.001. All values are presented as Mean ± SEM (n = 5-6 for in vivo and n = 3 biological replicates for in vitro experiments). Combo 2: Combinatorial treatment with OL, HT, OC; DAPI: 49,6‐diamidino‐2‐phenylindole; DHE: dihydroethidium DMSO; Dimethyl sulfoxide; HT: Hydroxytyrosol; LDL: Low density lipoprotein; MDA: malondialdehyde OC: Oleocanthal; OL: Oleuropein; ORO: Oil Red O; TBARs: thiobarbituric acid reactive substances. (For interpretation of the references to colour in this figure legend, the reader is referred to the Web version of this article.)

Taking into consideration the potent antioxidant properties of Combo 2, we hypothesized that Combo 2 and its bioactive constituents could alleviate NETosis, which is a critical event driving atherosclerosis development requiring the participation of ROS [74,75]. HT at both doses and OC at high dose (50 μg/ml) significantly inhibited NETosis after PMA activation, compared to the activated neutrophils without these compounds (Fig. 7A, B and Supplementary Table 8). Because of the vast inhibition caused by HT alone, Combo 2 was only tested in a lower dose in order to enable the revelation of any additive effects. No cumulative or synergistic effects of the constituents were observed on NETosis inhibition. When incubated alone, without PMA activation, the isolated compounds, as well as their combination, did not cause NETosis compared to control (Fig. 7A, B and Supplementary Table 8).

Based on the favorable effects of the constituents on NETosis, we aimed to evaluate whether Combo 2 or its constituents possess anti-atherosclerotic properties. Aortic atheromatic plaques from ApoE^−/−^ mice receiving WD for 12 weeks were evaluated in terms of lesion area to detect any effect of the isolated compounds or their mixture on the plaque development. Pretreatment with 10.13039/100014246HT, OC or Combo 2 significantly reduced the Oil Red O positive area (Fig. 7D and E) and the ROS formation in the aortic segments as indicated by the reduction in DHE relative fluorescent intensity (Fig. 7F and G) supporting the link between oxidative stress alleviation and the anti-atheromatic properties of the compounds and their combination. Additionally, HT, OC or Combo 2 reduced MDA levels while only HT and Combo 2 reduced the ox-LDL levels in the circulation of ApoE^−/−^ mice after 4-weeks pretreatment indicating again the superiority of Combo 2 compared to the isolated compounds (Fig. 7H and I). None of the treatments reduced cholesterol levels (Fig. 7J) indicating that the anti-atheromatic effect is linked to oxidative stress alleviation and not to a lipid-lowering potential.

### 3.8 Multi-organ toxicity evaluation of Combo 2

Finally, to ensure the safety of the combination therapies, we aimed to assess the potential subacute multi-organ toxicity of Combo 2 *in vivo* for 4 weeks at the previously selected dose and in a potentially toxic dose, which was three times higher than the previously employed dose. No significant differences were observed in serum biomarkers of liver toxicity [Aspartate Aminotransferase (AST), Alanine Aminotransferase (ALT)], kidney toxicity (Creatinine, Urea), or tissue damage [Lactic Dehydrogenase (LDH), Alkaline Phosphatase (ALP)] in Combo 2-treated groups compared to Veh (DMSO 5 %) group. Blood cell count, hematocrit and hemoglobin were not significantly changed by treatment with Combo 2, indicating absence of hematologic toxicity (Supplementary Table 9). Histological evaluation also showed no signs of injury or inflammation in heart, liver, kidney, pancreas, small intestine and lung tissues (Supplementary Fig. 9). These findings suggest that treatment with the combinatorial treatment does not induce toxicity in healthy animals even in doses higher than the effective, depicting a safe toxicity profile.

### 3.9 Clinical findings

After confirming that Combo 2 presents a safe toxicity profile *in vivo* even in a dose three times higher than the effective one, we aimed to test whether its beneficial effects could be translated into the clinical setting. 15 patients of both sexes were recruited with average age of 60.2 ± 8.9 years old. Their demographic and clinical characteristics are presented in Supplementary Table 10. The clinical study design is depicted in Supplementary Fig. 4.

Evaluation of vascular function showed significantly lower PWV, PBR_20-25 (μM)_ and higher FMD after administration of the supplement compared to baseline (p < 0.05). Additionally, Coronary Flow Reserve (CFR) was improved after administration of the supplement (p < 0.001) but not after placebo (p = 0.717). No significant differences were observed between the Placebo and Baseline measurements (Table 1). Improved LV function was observed after treatment with the supplement compared to baseline, as indicated by the reduced Deceleration time (DT) and E/e’ wave ratio, improved Left Ventricular Longitudinal Global Strain (LVGLS) and increased E’ wave/velocity (p < 0.05) (Table 2).

**Table 1:** Vascular biomarkers as determined at baseline, after one month of placebo administration and after one month of supplement treatment.

Table 1	Baseline	Supplement	Placebo	P^a^ (Suppl vs Baseline)	p^b^ (Placebo vs Baseline)
PWV (m/sec)	12.7 ± 1.3	12.1 ± 1.4	12.5 ± 1.2	0.034^c^	0.214
Aix (%)	13.1 ± 24.4	23.1 ± 9.9	32.3 ± 34.9	0.195	0.104
cSBP (mmHg)	125.5 ± 19.5	122.4 ± 18.1	124.2 ± 17.1	0.430	0.697
cDBP (mmHg)	77.2 ± 12.5	76.1 ± 9.1	78.1 ± 11.9	0.716	0.588
cPP (mmHg)	48.5 ± 15.4	46.7 ± 15.7	47.8 ± 14.9	0.189	0.443
PBR_5-25 (μm)_	2.34 ± 0.16	2.33 ± 0.19	2.37 ± 0.17	0.865	0.521
PBR_5-9 (μm)_	1.26 ± 0.13	1.30 ± 0.16	1.33 ± 0.13	0.404	0.141
PBR_10-19 (μm)_	2.48 ± 0.18	2.48 ± 0.21	2.51 ± 0.15	1.000	0.633
PBR_20-25 (μm)_	3.09 ± 0.34	2.93 ± 0.24	3.07 ± 0.29	0.028^c^	0.801
brSBP (mmHg)	127.4 ± 23.4	126.1 ± 22.4	125.1 ± 19.1	0.752	0.545
brDBP (mmHg)	71.3 ± 12.9	70.4 ± 13.9	72.5 ± 16.1	0.794	0.714
FMD (%)	3.22 ± 1.75	5.90 ± 2.47	3.08 ± 1.08	<0.001^d^	0.591
CFR	2.45 ± 0.46	2.73 ± 0.50	2.43 ± 0.47	<0.001^d^	0.717

Aix: Augmentation Index; brDBP: brachial diastolic blood pressure; brSBP: brachial systolic blood pressure; CFR: Coronary Flow Reserve; cSBP: central systolic blood pressure; cDBP: central diastolic blood pressure; cPP: central pulse pressure; FMD: flow-mediated dilation; PBR5-25 (μm): perfused boundary region of the sublingual arterial microvessels ranged from 5 to 25 μm; PWV: Pulse Wave Velocity. Values are presented as Mean ± SEM.

a Comparison Supplement vs baseline.

b Comparison Placebo vs baseline.

c p < 0.05.

d p < 0.0001.

**Table 2:** Echocardiographic parameters assessed at baseline and after one-month placebo or supplement treatment.

Table 2	Baseline	Supplement	Placebo	P^a^ (Suppl vs Baseline)	p^b^ (Placebo vs Baseline)
LVEDV (ml)	108.5 ± 31.8	105.1 ± 32.1	111.6 ± 33.6	0.492	0.375
LVESV (ml)	52.6 ± 19.7	53.8 ± 20.1	51.8 ± 19.8	0.522	0.430
LVEDD (mm)	49.8 ± 5.9	47.6 ± 7.1	48.3 ± 6.2	0.373	0.680
LVESD (mm)	36.8 ± 8.4	35.4 ± 8.8	36.1 ± 8.2	0.218	0.800
EF (%)	57.4 ± 22.1	56.9 ± 21.3	55.8 ± 20.3	0.669	0.727
LVMi (g/m2)	82.6 ± 25.8	83.6 ± 27.1	84.5 ± 24.5	0.727	0.823
IVSd (mm)	9.4 ± 1.5	9.5 ± 1.6	9.4 ± 1.4	0.843	0.952
PWd (mm)	8.8 ± 1.4	9.1 ± 1.5	8.9 ± 1.4	0.654	0.783
RWT	0.32 ± 0.06	0.33 ± 0.06	0.33 ± 0.07	0.411	0.758
LAd (mm)	42.5 ± 5.6	43.8 ± 4.9	43.4 ± 5.4	0.198	0.305
LA ESV BP index (ml/m2)	33.7 ± 10.6	33.3 ± 11.9	33.8 ± 12.1	0.953	0.797
E (cm/sec)	68.5 ± 14.5	72.1 ± 15.7	74.5 ± 16.5	0.431	0.272
A (cm/sec)	77.2 ± 13.5	72.4 ± 17.8	75.3 ± 15.5	0.245	0.332
DT (msec)	185.4 ± 65.3	156.8 ± 63.1	174.9 ± 58.6	0.020^c^	0.592
E’ average (cm/sec)	8.4 ± 2.8	9.8 ± 1.8	9.1 ± 2.1	0.032^c^	0.165
E/e’	8.4 ± 2.7	7.1 ± 2.1	8.1 ± 2.3	0.040^c^	0.421
LVGLS (%)	−17.3 ± 3.2	−18.1 ± 2.7	−17.7 ± 2.4	0.004^d^	0.031^b^

LVEDV: Left Ventricular End Diastolic Volume, LVESV: Left Ventricular End Systolic Volume, LVEDd: Left Ventricular End-Diastolic Diameter, LVESD: Left Ventricular End-Systolic diameter, EF: Ejection Fraction, LVMi: Left Ventricular Mass index, IVSd: Interventricular septum thickness at end diastole, PWd: Posterior wall thickness at end diastole, RWT: Relative Wall Thickness, LAD: Left Atrial Diameter, LA ESV BP index: Left Atrial End-Systolic Volume index, DT: Deceleration time, TDI: Tissue Doppler Imaging, e’: average lateral and septal velocity of mitral annulus, E: early mitral inflow velocity by Doppler, Α: late mitral inflow velocity, during atrial contraction, LVGLS: Left Ventricular global Longitudinal Strain. Values are presented as Mean ± SEM.

a Comparison Supplement vs Baseline.

b Comparison Placebo vs Baseline.

c p < 0.05.

d p < 0.01.

The supplement caused a significant reduction in proprotein convertase subtilisin/kexin type 9 (PCSK9), ox-LDL and MDA levels compared to Baseline measurements implying a potent antioxidant effect (Table 3).

**Table 3:** Biochemical serum parameters at baseline and after one-month treatment with placebo or supplement.

Table 3	Baseline	Supplement	Placebo	P^a^ (Suppl vs Baseline)	p^b^ (Placebo vs Baseline)
CRP (mg/L)	3.23 ± 1.81	1.96 ± 1.78	2.81 ± 1.77	0.038	0.571
PCSK9 (ng/mL)	239.2 ± 25.3	165.2 ± 21.3	205.7 ± 24.3	0.024^c^	0.135
Ox-LDL (ng/mL)	162.7 ± 36.5	146.9 ± 41.1	158.4 ± 38.3	0.011^c^	0.784
MDA (mmol/lt)	1.62 ± 0.35	1.41 ± 0.38	1.48 ± 0.42	0.046^c^	0.132
3-NT (ng/mL)	32.7 ± 6.8	35.1 ± 7.2	32.2 ± 6.9	0.167	0.486
Lp(a) (mg/dL)	3.27 ± 1.5	3.11 ± 1.54	3.16 ± 1.48	0.506	0.277

CRP: C-Reactive Protein, PCSK9: Proprotein Convertase Subtilisin/Kexin Type 9, OxLDL: Oxidized Low-Density Lipoprotein, MDA: malondialdehyde, 3-NT: 3- Nitrotyrosine, LP-a: lipoprotein -a. Values are presented as Mean ± SEM.

a Comparison Supplement vs Baseline.

b Comparison Placebo -vs Baseline.

c p < 0.05.

Finally, we sought to explore the potential enhanced benefit of the studied supplement enriched in OL, HT and OC, in comparison to the HT-enriched olive oil supplement that we have previously studied [60]. Statistical analysis revealed more potent beneficial effects by the OL, HT, OC-enriched supplement on the coronary function marker CFR, the endothelial glycocalyx parameter PBR_20-25 (μm)_ and the myocardial deformation marker LVGLS (p < 0.05). Furthermore, the OL, HT, OC-enriched supplement induced a significantly larger reduction in PCSK9 and ox-LDL compared to the HT-enriched olive oil (p < 0.05) (Table 4).

**Table 4:** Comparison between the changes in vascular, echocardiographic and biochemical parameters exerted by the HT-supplemented Olive Oil previously studied by our group and the OL, HT, OC- supplemented Olive Oil studied in the present work.

Change from Baseline	HT-Suppl Olive Oil	OL, HT, OC- Suppl Olive Oil	P value
PWV (m/sec)	−0.31 ± 0.25	−0.32 ± 0.27	0.799
CFR	0.21 ± 0.18	0.31 ± 0.14	0.035^a^
FMD (%)	2.58 ± 1.45	2.68 ± 1.51	0.935
PBR_5-25 (μm)_	−0.14 ± 0.12	−0.06 ± 0.14	0.204
PBR_20-25 (μm)_	−0.09 ± 0.19	−0.16 ± 0.23	0.045^a^
e’ average (cm/sec)	1.11 ± 2.25	1.17 ± 2.43	0.553
E/e’	−1.2 ± 0.05	−1.3 ± 0.03	0.645
LVGLS (%)	−0.34 ± 0.85	−0.74 ± 0.76	0.041^a^
PCSK9 (ng/mL)	−75.4 ± 21.4	−82.3 ± 16.2	0.038^a^
OxLDL (ng/mL)	−14.2 ± 13.4	−21.3 ± 11.2	0.025^a^
MDA (mmol/lt)	−0.37 ± 0.93	−0.26 ± 0.37	0.211

PWV: Pulse Wave Velocity, CFR: Coronary Flow Reserve; FMD: flow-mediated dilation; PBR_5-25 (μm)_: perfused boundary region of the sublingual arterial microvessels ranged from 5 to 25 μm; PBR_20-25 (μm)_: perfused boundary region of the sublingual arterial microvessels ranged from 20 to 25 μm; e’: average lateral and septal velocity of mitral annulus, E: early mitral inflow velocity by Doppler, Α: late mitral inflow velocity, during atrial contraction, LVGLS: Left Ventricular global Longitudinal Strain.; PCSK9: Proprotein Convertase Subtilisin/Kexin Type 9, OxLDL: Oxidized Low-Density Lipoprotein, MDA: malondialdehyde. Values are presented as Mean ± SEM.

a p < 0.05.

## 4 Discussion

In the present work, we aimed to design a combinatorial treatment of olive bioactive constituents with proven cardioprotective effectiveness and safety. Initially, we present that OL, OC and OA exerted significant infarct-size limiting properties, when administered in healthy animals. In order to increase the translational value of our study, we also evaluated their cardioprotective potential in animals with comorbidities, which could interfere with the mechanism of action of multiple cardioprotective interventions [76] and we found that OL, OC and OA significantly reduced infarct size, implying that the presence of confounding pathologies does not hinder their therapeutic potential.

We established a diet-induced MS model, as supported by the coexistence of obesity, hypercholesterolemia and hyperglycemia accompanied by glucose intolerance. These pathologies were present as early as the 8^th^ week of WD administration. Therefore, aiming to design a treatment possibly able to alleviate established confounding factors of CVDs, we selected this timepoint for the onset of oral administrations, adding translational value to our study. OL provoked an increase in circulating insulin which partially seems to contribute to the attenuation of hyperglycemia, in line with several approved antidiabetic pharmacological agents [77]. Moreover, OA reversed the dysregulation in lipidemic profile by restoring total cholesterol to similar levels to baseline. Even though reports have demonstrated anti-obesity effects of OL [78], various metabolic benefits of HT [19] and antidiabetic effects by OA [79], definitive differences in the experimental protocols (e.g. higher doses than the nutritional, treatment onset concomitantly with the induction of the corresponding pathology) might explain inconsistences in the reported results, compared to the findings herein presented.

Regarding the infarct size limiting potential of the olive derived compounds, we have previously described that OL attenuates myocardial IRI after acute administration during ischemia in different animal models at pharmacological doses [28] and chronic pretreatment with OL at a nutritionally relevant dose was cardioprotective in a rabbit hypercholesterolemic model of IRI, highlighting the potential of OL to demonstrate its protective effects in comorbid models [26]. Our results further strengthen these conclusions, as we showed that chronic pretreatment with OL in both healthy and MS burdened wild-type mice induces robust cardioprotection, in spite of all the modifications in molecular signaling caused by MS [80]. The employment of OL, as a well-established cardioprotective agent, reinforces the comparative aspect of our work, facilitating the distinction between protective and non-protective treatments. Preclinical studies on HT-induced cardioprotection are limited. Up to now only one *in vivo* study has presented the infarct size sparing and cardiac injury biomarkers reduction potential of HT. However the aforementioned study, contains major differences in the applied dose regimen and route of administration compared to our study, which might justify the discrepancy between the studies [81]. To the best of our knowledge, this is the first time that OC is investigated in the IRI setting. Interestingly, OC exerted cardioprotection both in presence and absence of MS. Lastly, OA administration was cardioprotective in healthy mice and in mice with MS in agreement with previous studies supporting its cardioprotective effects *in vivo* in normoglycemic and hyperglycemic rats [33] and in an isoproterenol-induced model of cardiac injury [40].

Aiming to combine cardioprotection and risk factor mitigation, we designed a combination therapy with possible additive protective effects in terms of mitigating cardiovascular disease risk factors and limiting the burden of major events, as we described above, and we found that Combo 2 presented the most favorable beneficial effects on IRI and MS parameters. In order to identify the cardioprotective mechanism of Combo 2 and its constituents, we firstly focused on apoptosis, a type of cell death that occurs mostly during reperfusion and represents a valuable target to impede the detrimental effects of myocardial IRI [82]. Indeed, all the treatments exhibiting infarct-size limitation (OL, OC, Combo 2), showed reduced apoptotic ratio (apoptotic Bax/antiapoptotic BcL-xL ratio). The inclusion of the non-cardioprotective HT in the analysis strengthened the hypothesis that apoptosis suppression participates in the mechanism of action responsible for the observed effect on infarct size. Activation of the RISK pathway has been associated with suppression of apoptotic signaling [83,84]. However, we did not observe any effect on the expression or phosphorylation of key-mediators GSK-3β and Akt [65]. A burst of ROS occurs at reperfusion and is a key regulator of myocardial infarct size and cell death [85]. Therefore, we speculated that the cardioprotective effects may be linked to their effect on oxidative stress, which is an important factor mediating IRI [82]. The expression levels of NOX-1/-2/-4 isoforms were shown to significantly increase infarct size with excessive ROS production [86]. In NOX-1, NOX-2 but not NOX-4 knockout mice a significant decrease in the size of myocardial infarct was observed following 30 min of ischemia and 24 h of reperfusion [87]. However, none of the treatments altered NOX isoforms’ expression indicating that the mechanism of cardioprotection of the isolated olive-derived constituents administered at a nutritional dose in mice with MS is not linked to ROS production by these enzymes. Redox-based treatment strategies for cardioprotection have been extensively studied [88,89], and MnSOD, CAT and HO-1 belong to the first-line antioxidant defense [90]. We observed a parallel increase of MnSOD and CAT by OC and Combo 2, which can be interconnected with enhanced antioxidant defense against IRI-derived ROS in the ischemic myocardium, whereas HO-1 expression remained unchanged by our treatments. Overexpression of the isoform MnSOD has been linked to reduced IRI [91,92]. Likewise, heart-specific CAT overexpression in transgenic mice has been associated with reduced IRI *ex vivo* [93]. Clearly, concomitant elevation of SOD and CAT offers a direct detoxification pathway of the free radicals burst during reperfusion, salvaging the myocardial tissue from subsequent cell death including apoptosis. In line with MnSOD and CAT alterations, we observed the upregulation of their transcription factor, Nrf2, by OC and Combo2. However, despite the large body of evidence describing the activation of Nrf2 by the olive oil constituents, the majority of the previously mentioned studies employed concentrations and doses far greater than those commonly found in the diet with EVOO [94]. In fact, we have previously shown that OL is cardioprotective when given acutely at reperfusion in higher dose and it upregulates Nrf-2 [28]. In our study, chronic OL administration in a nutritional dose did not affect Nrf2 and its downstream targets expression, a fact that can be attributed to the dose and timing of OL. OL alone did not affect oxidative stress markers, such as MDA and ox-LDL, further suggesting that the observed reduction in apoptosis may not be linked to oxidative stress. 3-NT levels which is a marker of nitro-oxidative stress, (generally obtained from the reaction between peroxynitrite (ONOO−) and tyrosine residue) were decreased by treatment with HT, OC and Combo 2 in the murine hearts further confirming the enhanced antioxidant effect of Combo 2 in comparison to the isolated bioactive constituents alone. This result suggests an antioxidant effect of these treatments but it may not be crucial for direct cardioprotection as we have previously shown for short-term statin treatment in hypercholesterolemia rabbits [95] since HT treatment does not alter infarct size. Regarding Combo2, cardioprotection was linked to oxidative stress reduction with decreased MDA and ox-LDL in the circulation, enhanced expression of MnSOD more than OC alone, CAT and Nrf2 and suppression of apoptotic death signaling and consequently protects the cardiac tissue from IRI.

The potent protection from oxidative stress in the heart, especially with the detrimental cellular alterations after an IRI, can also be applied to prevent other pathophysiologic processes. HT and OC inhibit NETosis by acting directly on the neutrophils. NETosis has been associated with the pathogenesis of atherosclerosis, contributing to multiple stages of the disease [96]. Also, pharmacological inhibition of NETosis resulted in alleviation of atherosclerotic plaque development [97]. HT, OC and Combo 2 can protect the LDL particles from oxidative modifications *in vitro* and *in vivo*, which represents a crucial step in atherogenesis *in vivo* [98]. Taken together, these effects may explain the antiatherosclerotic effect observed herein *in vivo,* as treatment with HT, OC or Combo 2 for 4 weeks daily significantly reduced the extent of atherosclerotic lesion areas and the levels of ROS formation in the aorta segments of ApoE^−/−^ mice.

Taking all the above into consideration, Combo 2 was the combinatorial treatment that pertained all the favorable effects of the isolated compounds (infarct size reduction, hyperglycemia attenuation and atherosclerotic plaque reduction), whereas showed superiority in the upregulation of antioxidant enzymes and in ROS suppression. In contrast, we found that coadministration of OL and OA eliminated their antihyperglycemic and anti-hypercholesterolemic effects respectively in Combos 1 and 3. After ruling out the possible chemical interaction in the treatment solution, we hypothesized that a counteracting pharmacological or pharmacokinetic interaction possibly occurs *in vivo*, which was not herein investigated. Consequently, our study highlights that potential interactions among constituents might negatively impact their beneficial capacity, which denotes the need for the standardization of nature-derived products as a prerequisite for their clinical applications. Paving the road towards their effective incorporation in preventive strategies, we need not only to identify which compounds show the most potent cardiovascular-protective effects, but also which combinations would pertain, the anticipated clinical results in presence or absence of cardiovascular comorbidities.

Considering the vast number of pharmacological agents failing to reach clinical benefit, despite the considerably encouraging preclinical data, we also performed a small-scale clinical study to ensure the beneficial impact of the olive constituent combination in very high-risk patients, namely patients with CCAS. We found that treatment with the supplement OL-HT-OC, which is translationally similar to the Combo 2 therapy used in our preclinical studies, conferred improved left ventricular, vascular and endothelial function in our study population. Furthermore, biomarkers linking oxidative stress and endothelial dysfunction (MDA, ox-LDL, PCSK9) showed significant reduction, after the 4-week supplement administration to the CCAS patients. MDA and ox-LDL levels reduction upon the OL-HT-OC-enriched supplement are in agreement with our animal studies further supporting the translational value of our data. The obtained results are similar to the effects of HT-supplemented olive oil in our previous work [60]. Since the two clinical studies share the same characteristics concerning study design and population, we compared the beneficial effects of the two treatments and we found that the OL-HT-OC supplement had greater impact on vascular parameters as well as biomarkers of oxidative stress and endothelial function compared to the supplement solely enriched in HT. Considering that endothelial function and integrity has been recently conceptualized as a major determinant of cardiovascular outcomes, a supplement based adjuvant endothelial protective therapy could be of particular benefit in high-risk populations [99]. In both clinical studies patients with diabetes were excluded but our data warrant future work to examine the effect of the OL-HT-OC enriched supplement in diabetic patients on a larger scale.

In the present work we sought to maintain high translational value in all our *in vivo* protocols, but as expected, some limitations exist. Our diet-induced MS model consisted of three specific pathologies and animals did not exhibit hypertension. Female mice are more resistant to the manifestations of MS [100,101] and for this reason in the present study, only male mice were used. Therefore, sex may stand as a limitation to the interpretation of the murine study results regarding the manifestations of metabolic syndrome and further studies using female animals with established MS should be conducted. However, recently it was demonstrated that infarct size does not differ between male and female Göttingen minipigs [102] suggesting that our findings regarding infarct size may be generalizable to both sexes. In the clinical setting both male and female patients were enrolled in the clinical study. Seasonal parameters may impact on the results interpretation, regarding the retrospectively comparative evaluation of the results of the infarct size, after administration of the isolated compounds and the combinatorial treatments. Although the experiments were carried out at different stages, the same environmental conditions were applied, and the same investigator conducted the experiments to minimize the experimental variation. As we focused on the compounds’ effects at nutritional doses, and because three out of the four compounds herein investigated, exerted infarct-sparing benefits in our primary preclinical endpoint at the selected nutritional doses, we did not perform dose-response experiments. HT failed to reduce infarct size, implying the need to test higher doses of the constituent, which was beyond the scope of our study. Nevertheless, we included two different doses of the investigated compounds in our multi-organ toxicity study to confirm the combination therapy safety. Lastly, in our clinical study we have recruited a small number of patients. Despite the low number of participants, we managed to acquire sufficient power to point out the beneficial effects of the selected olive constituents on the studied clinical parameters, which were greater than the benefits of the supplement enriched with HT, which was previously studied.

In conclusion, we designed a combinatorial treatment, consisting of olive-derived bioactive compounds at nutritional doses, which presents potent protective properties against IRI and atherosclerotic plaque development as well as antihyperglycemic effects. Additionally, our study provides the first evidence of investigating oleocanthal as a novel cardioprotective compound in future preclinical and clinical studies. Treatment of MS relies nowadays primarily on lifestyle and dietary modifications and in case of insufficient results may require specific medication for each individual risk factor, which significantly elevates the risk for major cardiovascular events and mortality. Focusing on the prevention strategy, our study provides novel translational data on a bench-to-bedside approach for patients with risk factors, paving towards the implementation of olive constituents and products as adjuvant nutraceuticals against the intricate cardiometabolic syndrome and IRI.

## Funding

This work was supported by the Operational Program “Competitiveness, Entrepreneurship and Innovation” of the General Secretariat for Research and Innovation, Hellenic Rebublic Ministry of Development, Greece under the call “RESEARCH – CREATE - INNOVATE” (project code: 5048539) and by a research grant from the 10.13039/501100010353Hellenic Atherosclerosis Society, Greece.

## CRediT authorship contribution statement

**Andriana Christodoulou:** Writing – original draft, Visualization, Methodology, Investigation, Formal analysis, Conceptualization. **Panagiota-Efstathia Nikolaou:** Writing – review & editing, Methodology, Investigation, Formal analysis. **Lydia Symeonidi:** Investigation. **Konstantinos Katogiannis:** Writing – review & editing, Investigation, Formal analysis. **Louisa Pechlivani:** Methodology, Investigation, Formal analysis. **Theodora Nikou:** Investigation. **Aimilia Varela:** Investigation, Formal analysis. **Christina Chania:** Investigation. **Stelios Zerikiotis:** Investigation. **Panagiotis Efentakis:** Methodology, Investigation. **Dimitris Vlachodimitropoulos:** Resources, Investigation. **Nikolaos Katsoulas:** Resources, Investigation. **Anna Agapaki:** Resources, Investigation. **Costantinos Dimitriou:** Resources, Investigation. **Maria Tsoumani:** Investigation, Conceptualization. **Nikolaos Kostomitsopoulos:** Resources. **Constantinos H. Davos:** Writing – review & editing, Resources, Formal analysis. **Alexios Leandros Skaltsounis:** Resources, Investigation. **Alexandros Tselepis:** Investigation, Formal analysis. **Maria Halabalaki:** Writing – review & editing, Resources, Investigation. **Ioulia Tseti:** Resources, Project administration, Funding acquisition. **Efstathios K. Iliodromitis:** Writing – review & editing, Project administration, Funding acquisition. **Ignatios Ikonomidis:** Writing – review & editing, Project administration, Investigation, Conceptualization. **Ioanna Andreadou:** Writing – review & editing, Supervision, Project administration, Funding acquisition, Conceptualization.

## Declaration of competing interest

The authors declare the following financial interests/personal relationships which may be considered as potential competing interests:Andreadou Ioanna has patent “Composition based on olive extract suitable for cardioprotection” pending to Uni-Pharma S.A. If there are other authors, they declare that they have no known competing financial interests or personal relationships that could have appeared to influence the work reported in this paper.

## Data Availability

Data will be made available on request.
