# Supplementary material for: Cardioprotective potential of oleuropein, hydroxytyrosol, oleocanthal and their combination: Unravelling complementary effects on acute myocardial infarction and metabolic syndrome
Source: Redox Biol. 2024 Aug 14;76:103311. doi: 10.1016/j.redox.2024.103311 (PMC11378258; doi:10.1016/j.redox.2024.103311)
Supplement: Multimedia component 1 [file mmc1.docx]

Supplementary Material

Cardioprotective potential of oleuropein, hydroxytyrosol, oleocanthal and their combination: Unravelling complementary effects on acute myocardial infarction and metabolic syndrome.

Andriana Christodoulou^a^, Panagiota-Efstathia Nikolaou^a^, Lydia Symeonidi^a^, Konstantinos Katogiannis^b^, Louisa Pechlivani^c^, Theodora Nikou^d^, Aimilia Varela^e^, Christina Chania^a^, Stelios Zerikiotis^a^, Panagiotis Efentakis^a^, Dimitris Vlachodimitropoulos^f^, Nikolaos Katsoulas^f^, Anna Agapaki^g^, Costantinos Dimitriou^h^, Maria Tsoumani^a^, Nikolaos Kostomitsopoulos^h^, Constantinos H. Davos^e^, Alexios Leandros Skaltsounis^d^, Alexandros Tselepis^c^, Maria Halabalaki^d^, Ioulia Tseti^i^, Efstathios K. Iliodromitis^j^, Ignatios Ikonomidis^b*^, Ioanna Andreadou^a*#^

^*^Contributed equally

*^a^ Laboratory of Pharmacology, Faculty of Pharmacy, National and Kapodistrian University of Athens, Panepistimioupolis, Zografou, Athens, Greece.*

*^b^ Laboratory of Echocardiography and Preventive Cardiology, Second Cardiology Department, Attikon University Hospital, National and Kapodistrian University of Athens, Medical School, Athens, Greece.*

*^c^ Atherothrombosis Research Centre/Laboratory of Biochemistry, Department of Chemistry, University of Ioannina, Ioannina, Greece.*

*^d^ Division of Pharmacognosy and Natural Products Chemistry, Department of Pharmacy, National and Kapodistrian University of Athens, Athens, Greece.*

*^e^ Cardiovascular Research Laboratory, Biomedical Research Foundation Academy of Athens (BRFAA), Athens, Greece.*

*^f^ Laboratory of Forensic Medicine and Toxicology, Medical School National and Kapodistrian University of Athens, Athens, Greece.*

*^g^ Histochemistry Unit, Biomedical Research Foundation, Academy of Athens* *(BRFAA), Athens, Greece*

*^h^ Centre of Clinical Experimental Surgery and Translational Research, Biomedical Research Foundation of the Academy of Athens (BRFAA), Athens, Greece.*

*^i^ Uni-Pharma S.A., Athens, Greece*

*^j^ National and Kapodistrian University of Athens, Medical School, Athens, Greece*

**^#^Corresponding author**: Prof Ioanna Andreadou, Laboratory of Pharmacology, Faculty of Pharmacy, Panepistimiopolis, Zografou, Athens 15771, Greece; tel: +30 210 7274827; fax: +30 210 7274747; e-mail: [jandread@pharm.uoa.gr](mailto:jandread@pharm.uoa.gr)

**Reagents for compounds isolation and purification**

All solvents were of analytical grade i.e n-Hexane (n-Hex), ethyl acetate (EtOAc), ethanol (EtOH) and acetonitrile (ACN) and were purchased from Carlo Erba Reactifs SDS (Val de Reuil, France) while methanol (MeOH) and dichloromethane (DCM) as well as sulfuric acid (H_2_SO_4_, > 95 %) were purchased from Fisher Scientific (Leicestershire, United-Kingdom). Deionized water was used to prepare all aqueous solutions. MeOH and ACN of HPLC gradient grade (Carlo-Erba) while ultra-purity water from Mili-Q device were used for HPLC-DAD analysis. Vanillin (98% purity) was purchased from Sigma-Aldrich (United-Kingdom).

**Isolation and purification of hydroxytyrosol, oleocanthal and oleanolic acid**

In order to isolate the target compounds a Fast Centrifugal Partition Extractor – FCPE was initially used for biophenols recovery from EVOO or TPF and then a Fast Centrifugal Partition Chromatograph - FCPC for the fractionation of TPF. Both devices were purchased from the Rousselet-Robatel Kromaton (Anonay, France). The FCPE300® device is equipped with a rotor composed of 7 stacked partition disks with 231 partition cells (≈ 1 mL per twin cell) with a total volume of 303 mL. The rotational speed can be adjusted from 500 to 2000 rpm. The solvents were pumped with a Knauer Preparative 1800 V7115 pump (Berlin, Germany) and fractions were collected by a Pharmacia Superfrac collector (Uppsala, Sweden). The “mobile” or feed phase (17.5 L) was prepared by mixing 10.5 L of n-Hex and 7 L of EVOO (ratio 3:2 v/v). The extracting or “stationary” aqueous phase (3 L) was prepared with 1.8 L of EtOH and 1.2 L of H_2_O (ratio 3:2 v/v). The FCPE column was filed at a flow rate of 75 mL/min and 200 rpm in the ascending mode with the extracting aqueous phase. Afterwards the rotation speed was increased to 1000 rpm and the mobile phase (EVOO and n-Hex) of 2.5 L was fed into the column at a flow rate of 60 mL/min for the extraction. Next, the pumping mode was switched to descending mode and fresh aqueous phase (0.3 L) was pumped at 60 mL/min and 1000 rpm. The extracting phase containing phenolic compounds (TPF) was recovered. This extraction cycle was repeated 7 times and all extracts were combined and evaporated to dryness under vacuum at 40° C and stored at 4° C until use. 7 cycles of extraction-extrusion were performed in about 6 hours corresponding to 17.5 L of organic phase, containing 7 L of EVOO. Thus, approximately 10 g of TPF were finally yielded. On the other hand, the FCPC1000® rotor is equipped with 45 circular partition disks and 32 partition cells (555 μL per cell) reaching a total capacity of 955 mL. The rotational speed can be adjusted from 200 to 2000 rpm. For mobile phase pumping a Prep 36 LabAlliance dual piston pump (State College, PA, USA) was used equipped with a PEEK preparative injector 3725 (Rheodyne, Rohnert Park, CA, USA) and a 30 mL sample loop. The system was hyphenated to a UV detector SPECTRA SYSTEM UV 2000, Thermo Scientific (Illkirch, France) and a fractions collector Büchi B-684 collector (Flawil, Switzerland). For fractionation of TPF and isolation purposes a preparative CPC column (1000 mL) was used. In total, 4 biphasic systems were used using the same type of solvents i.e. n-hex/EtOAc/EtOH/H_2_O in different analogies. The column was initially filled with the lower aqueous phase of S1 (n-hex/EtOAc/EtOH/H_2_O: 4/1/2/3) at a flow rate of 30 mL/min and the rotation speed was set to 300 rpm, in the ascending mode. Afterwards, the rotation was set at 900 rpm and the first mobile phase corresponding to the organic phase of S1 was pumped (flow rate of 20 mL/min). After hydrodynamic equilibrium the sample (5g of TPF in 30mL of S1) was injected. Then, a sequential pumping of the upper mobile phases of S1, S2 (3/2/2/3), S3 (2/3/2/3) and S4 (1/4/2/3) took place in volumes of 500 mL, 1100 mL, 1400 mL and 1000 mL respectively (elution phase). Next, the system was switched to ‘‘descending’’ mode and 750 mL of the lower phase of S4 were fed (extrusion phase). Fractions were collected every 25 mL and the entire procedure lasted 240 min. In total 190 fractions were obtained. All fractions were analyzed by TLC and were pooled affording finally 15 combined fractions.

In order to achieve high purity of the target compounds silica gel and/or size exclusion chromatography techniques were employed. Certain combined CPC fractions 2-5 (554 mg), 18-26 (420 mg) and 126-140 (342 mg) were further analyzed using low pressure column chromatography. As stationary phase normal phase Silica gel 60H® (0.04-0.06 mm) was used and mixtures of DCM, EtOAc and MeOH in increasing polarity mode were used for the elution (i.e. DCM 100%, DCM/EtOAc 98/2 - 96/4 - 92/8 - 84/16 - 68/32 - 36/64 v/v, EtOAc 100%, EtOAc/ MeOH 50/50 v/v). All collected fractions (10 mL) were monitored qualitatively by TLC. The combined CPC fractions 44-52 (175 mg) and 80-84 (114 mg) were analyzed using size exclusion column chromatography with Sephadex LH-20® as stationary phase and compounds were eluted with EtOH. Similarly, all fractions were monitored by TLC. The TLC analysis was performed on Merck 60 F254 pre-coated silica gel plates and developed with DCM/MeOH in various analogies i.e. 95:5, 90/10, 80/20, 70/30 (v/v) and monitored under UV light at 254 and 365 nm. TLC plates were then sprayed by a vanillin (5% w/v in ethanol) – H_2_SO_4_ (50% v/v in methanol) solution and heated at 100–120^0^C for 2–3 min.

**Isolation and purification of oleuropein**

Initially, 320 gr of dry leaves after pulverization were extracted using ultrasounds operating at a frequency of 37 kHz (Elmasonic S 100H, Elma, Germany) with acetone affording 28 gr of dry extract. After defatting with CH_2_Cl_2_/MeOH:98/2 (v/v), 6 gr of final extract was obtained enriched in OL. For separation and isolation of OL, the FCPC technique was used similarly to the other target compounds but following a different methodology according to Boka and coworkers [1] due to the different starting material. The instrumentation used was the same but a single biphasic solvent system i.e. n-Hex/EtOAc/EtOH/H_2_O 1/9/1/9 (v/v/v) was used. Briefly, the column was fed with the upper organic phase as the stationary phase. Then, the rotation was set at 900 rpm and the lower more polar phase was pumped at a flow rate of 30 mL/min in the descending mode. When the hydrodynamic equilibrium was reached, the defatted extract was injected. Afterwards, fractions were collected and monitored by TLC as described above for HT, OC and OA. For further purification of OL, a preparative HPLC was employed. More specifically a Thermo Finnigan HPLC system (Ontario, Canada) an UV SpectraSystem UV6000LP detector monitored at 235, 280 and 365 nm, was used equipped with a chromatographic column (Discovery HS C18, 250 mm × 10 mm, 5 μm, Supelco, Sigma). For elution, a simple gradient system of ACN/H_2_O (5/95 to 40/60) in 30 min was employed and monitoring under 250 nm.

**Compounds’ structure verification and purity determination**

All the isolated and purified compounds were analyzed by nuclear magnetic resonance (NMR) and High-Resolution Mass ([HRMS](https://www.sciencedirect.com/topics/chemistry/high-resolution-mass-spectrometry-hrms)) spectrometry for structure verification (Supplementary Fig. 1A-D). ^1^H, ^13^C (1D-2D) NMR experiments were performed on a 600 MHz Bruker Avance AVIII-600 spectrometer (Karlsruhe, Germany) equipped with a TXI cryoprobe (Wissembourg, France) in CDCl_3_ (OC, OA) and CD_3_OD (HT, OL). For HRMS an Orbitrap Discovery Mass Spectrometer (Thermo Scientific, Brehmen, Germany) was employed. The mass spectrometer was equipped with [electrospray ionization](https://www.sciencedirect.com/topics/chemistry/electrospray-ionization) (ESI) source and operated in negative mode.

Moreover, the target compounds were analysed by HPLC-PDA for purity determination apart from OA due to its low absorbance under UV light (Supplementary Fig. 1E). The method used is proposed by International Olive Council (IOC) for the analysis of olive polyphenols [2]. The analytical system used is consisted of SpectraSystem SCM1000 mobile phase solvent degasser, SpectraSystem P4000 pump, with the ability to mix four different solvents, in any ratio, SpectraSystem AS3000 autosampler, SpectraSystem UV6000LP photodiode array detector (PDA) from Thermo Finnigan and a Spherisorb ODS-2 (C18), (250 x 4.6 mm), 5μm chromatography column. The mobile phase consisted of the solvents: A) 0.2% aqueous phosphoric acid solution, B) ACN:MeOH mixture (50:50v/v). Separation was achieved by a gradient elution system as follows: starting conditions 96% A (T=0); in 40 min 50% A; in the next 5 min 40%A (T=45 min); in the next 15 min, 0%A which maintained for additional 10 min (T=70 min); return to initial conditions in 2 min and finally conditioning for additional 10 min at 96% A. The mobile phase flow was set at 1ml/min, the temperature at 20°C and the injection volume at 20 µL. The detection was carried out at 280 nm and the purity determination was carried out by comparing with reference standards. ChromQuest software (Chromatography Data System Software) was employed for acquisition as well as data handling and interpretation. Finally, HT (98.5%, HPLC-DAD), OL (98% purity, HPLC-DAD), OC (98.5% purity, HPLC-DAD) and OA (98%, NMR) were obtained. The entire procedure was repeated several times when necessary to meet the quantity requirements for the *in vivo* experiments.

**Power analysis for sample size determination**

Aiming to obtain reliable results using the minimum number of animals required, we performed *a priori* power analysis using the software GPower Analysis 3.1.9.4. The infarct size, determined as the percentage of necrotic area within the area at risk (Infarct size/ Area at risk %), was selected as the primary endpoint. F-Test ANOVA: fixed effects, omnibus, one-way was applied with α-error probability set at 0.05, power at 0.80 and effect size was calculated from mean values. For the 1^st^ experimental series, expected infarct size mean of each group was estimated based on literature references implementing the same or similar experimental protocols and dose regimens as follows: Vehicle group (DMSO 5%) 36% [3], oleuropein (OL) 23% [4], hydroxytyrosol (HT) 18% [5], oleanolic acid (OA) 36% [6]. To the best of our knowledge, oleocanthal (OC) is being studied for the first time in the IRI setting, but because of its the potent anti-inflammatory and antioxidant effects we also anticipated cardioprotection (OC 18%). Therefore, the minimum required number of animals was calculated at n=4 per group. For the 2^nd^ experimental protocol, we expected that vehicles do not affect the studied parameters and the isolated compounds would confer the same effects in both the MS and healthy animals, so the mean infarct size values obtained in the 1^st^ experimental series was used as the expected mean value for the 2^nd^ experimental series. The minimum required number of animals was n=6. Lastly, for the 3^rd^ experimental protocol the estimated mean values were based on the results obtained from the 2^nd^ series of experiments, taking into consideration the infarct size limiting effect exerted by the most effective cardioprotective constituent. N=6 animals per group are essential to achieve enough power to compare the primary endpoint among groups.

SDσ within groups was set at 8 according to previous studies implementing the same experimental protocol of left anterior descending (LAD) artery ligation for 30 minutes followed by 2-hour reperfusion to induce myocardial infarction. Due to the severity of the surgical intervention, we also calculated a 10% dropout rate [7]. Animals that did not complete the 30 minutes of ischemia and the 2-hour reperfusion were excluded from measurements, as the ischemia/reperfusion injury evolves in a time manner and different ending timepoints eliminate the comparative aspect of our study.

**Fasting glucose and glucose tolerance test.**

Blood glucose was determined by analyzing a drop of blood from the tail vein of 14-hours fasted animals, using a glucose meter (Accu chek instant). Glucose Tolerance Test (GTT) was performed as previously described [8]. After the first measurement of fasting glucose, each animal received intraperitoneally 2 g/kg glucose solution [D-(+) Glucose (Sigma, #G7021) diluted in sterile normal saline (NaCl 0,9%)] and blood glucose was monitored at predetermined timepoints: 15, 30, 60, 90, 120, 180 minutes. Construction of the glucose curve enabled the calculation of the AUC which was compared among groups [9].

**Insulin, toxicity biomarkers and lipid profile in plasma/serum**

Plasma was collected using heparin diluted 1:6 in water for injection as anticoagulant (heparin:blood, 1:10 v/v) and centrifuging at 5000 x g for 15 minutes. For toxicity evaluation (6^th^ experimental series) we received serum by allowing blood to clot at room temperature and centrifuging at 3000 x g for 15 minutes. Insulin was determined in plasma using an ultrasensitive enzyme-linked immunosorbent assay (ELISA; Cat. No. 10-1132-01; Mercodia, Uppsala, Sweden) after 14 hours of fasting. Homeostatic model assessment for insulin resistance (HOMAIR) was calculated as follows: HOMAIR = [Fasting Insulin (μIU/ml)* Glucose (mmol/L)]/22.5. Quantification of Total cholesterol (#000218), Triglycerides (#000243), Aspartic Aminotransferase (AST) (#1695), Alanine Aminotransferase (ALT) (#001696), Lactic Dehydrogenase (LDH) (#001698), Alkaline Phosphatase (ALP) (#001694), Creatinine (#000216), Urea (#001526) was performed using commercially available assay kits purchased by Biotecnological Applications Ltd and all measurements were performed per manufacturers' instructions. Blood was analyzed by an Automated Hematology Analyzer (Sysmex pocH 100iV Diff) to determine the number of cells (White Blood Cells, Red Blood Cells and Platelets), Hematocrit and Hemoglobin

**Western blot**

Sample preparation and Western blot analysis were performed as previously described [10,11]. Protein content was isolated from the ischemic heart tissue using RIPA Lysis Buffer (1% Triton X-100, 20 mM Tris pH 7.4-7.6, 150 mM NaCl, 50 mM NaF, 1 mM EDTA,1 mM EGTA, 1 mM Glycerolphosphatase, 1% SDS, 100 mM phenylmethylsulfonyl fluoride, and 0.1% protease phosphatase inhibitor cocktail), after centrifugation at 11000 x g, 15 min, 4 °C). Protein was quantified by Lowry assay in the supernatants and samples with the same protein load were prepared using Dave’s buffer (4% SDS, 10% 2-mercaptoethanol, 20% glycerol, 0.004% bromophenol blue, and 0.125 M Tris·HCl). Protein samples were electrophoretically separated by 10% SDS-PAGE along with a colored protein ladder (Nippon Genetics BlueStar Plus Prestained Protein Marker), transferred on polyvinylidene difluoride (PVDF) membrane (Cytiva Amersham™ Hybond™ P 0.45μm PVDF Membrane) and immunoblotted with primary antibodies for: Bax (Rabbit mAb #14796, dilution 1:1000), B-cell lymphoma-extra large (BcL-xL) (Rabbit mAb #2764, dilution 1:1000), Glycogen Synthase Kinase 3 beta (GSK-3β) (Rabbit mAb #9315), phospho-Protein Kinase B Ser473 (p-Akt) (Rabbit mAb #4060, dilution 1:1000), Protein Kinase B (t-Akt) (Rabbit pAb #9272, dilution 1:1000), Manganese-superoxide dismutase (MnSOD) (Rabbit mAb #13141, dilution 1:2000), Heme-oxygenase-1 (HO-1) (Rabbit mAb #43966, dilution 1:1000), Nuclear factor erythroid 2-related factor 2 (Nrf2) (Rabbit mAb #12721, 1:500), NADPH oxidase cytosolic protein (p47phox) (Rabbit mAb #63290, 1:1000), Nitro-Tyrosine Antibody (Rabbit Ab #9691, dilution 1:500), α-Actinin (Rabbit mAb #6487, dilution 1:2500), Glyceraldehyde-3-phosphate dehydrogenase (GAPDH) (Rabbit mAb #2118, dilution 1:2500) purchased from Cell Signaling Technology via Bioline Scientific, Catalase (CAT) (Mouse mAb, #sc-271803, dilution 1:10000), phospho-Glycogen Synthase Kinase 3 beta Ser9 (GSK-3β) (Mouse mAb #sc-373800, dilution 1:1000) and heme binding subunit of the superoxide-generating NADPH oxidase (gp91-phox) (Mouse mAb #sc-130543) purchased from Santa Cruz Biotechnology and NADPH oxidase 1(NOX-1) (Rabbit Ab#DF8684, dilution 1:1000) purchased from Affinity Biosciences via BioInnotech. Finally, membranes were incubated in the corresponding Horseradish Peroxidase (HRP)-conjugated secondary antibody (either Anti-rabbit IgG, HRP-linked Antibody #7074, dilution 1:2000, or Anti-mouse IgG, HRP-linked Antibody #7076, dilution 1:2000) purchased from Cell Signaling Technology. Binding of the secondary antibody allows protein detection by chemiluminescence captured by ImageQuant LAS 500 after exposure to the HRP substrate, ECL (Immobilon Forte Western HRP substrate, Millipore). Relative densitometry analysis was performed using GelPro Analyzer 4.0 software. GAPDH and α-Actinin served as loading control proteins. For quantification graphs, all values were normalized to the respective vehicle-treated group densitometric measurements [7]. Uncropped Western blots are depicted in Supplementary Figures 10-12.

***In vitro* TBARS method**

The potential inhibitory effect of the most effective combinatorial treatment, Combo 2, or its isolated constituents on Low Density Lipoprotein’s (LDL) oxidative modification was evaluated by thiobarbituric acid reactive substances (TBARS) method as previously described [12]. Low Density Lipoprotein from human plasma (lyophilized powder, Sigma #L8292) was diluted in PBS 1x to prepare a 200 μg/ml solution. 500 μL LDL solution were incubated for 10 minutes with one of the following treatments: i. Vehicle (DMSO 5%), ii. OL, iii. HT, iv. OC, v. Combo 2. All treatments were added at a final concentration of 50 μM for comparative reasons. The LDL mixture was, then exposed to 1mM Copper (II) sulfate (CuSO_4_) (Sigma #451657) in double-distilled water for 6 hours on shaking platform at 37^o^C. Oxidation was stopped by adding 30 μL Ethylene diamine tetraacetic acid (EDTA) 1 mM and incubation for 5 minutes at room temperature. 2-Thiobarbituric Acid (TBA) (Sigma #T5500) solution 0.05 M was prepared by diluting the appropriate amount of TBA powder in 10% DMSO in double distilled water at 50°C for 45 minutes. 300 μL of the incubation mixture were received in a new tube with 600 μL TBA. Samples were then heated at 100^o^C for 15 minutes to promote the formation of a colored complex of oxidation end-products and centrifuged at 10000 x g for 10 minutes. Lipid peroxidation products were quantified in supernatants spectrophotometrically by measuring the absorbance at 490 nm. Lipid peroxidation products were expressed as malondialdehyde (MDA) equivalents so TBARS concentration was calculated using the molar extinction coefficient of MDA“ (156,000 (L/mol/cm)) [12].

**Compounds’ stability in combinatorial treatment solution- NMR analysis.**

The chemical stability of OL and OA in mixtures was assessed by Nuclear Magnetic Resonance (NMR) spectroscopy. OL and OA standards were diluted in deuterated methanol (MeOD) in the concentrations ratio used *in vivo* (OL:OA 1:0.84). Sample solutions were analyzed by ^1^Η-NMR (Supplementary Tables 5, 6 and Supplementary Fig. 7). Then the 2 solutions were combined, the mixture was concentrated until dry and resuspended in 600 μL MeOD. The mixture was analyzed under the same conditions as the standard solutions in order to compare the spectra of each compound before and after mixing (Supplementary Fig. 7). Analysis was conducted using 600 MHz Bruker Avance AVIII-600.

**Clinical study variables**

Endothelial Function

The assessment of brachial artery flow-mediated dilation (FMD) followed established methodology [13]. Values are calculated as the percentage increase from the baseline arterial diameter. The intra-observer variability for brachial artery diameter was 0.1 ± 0.12 mm.

Endothelial Glycocalyx

We utilized Sidestream Darkfield Imaging (Microscan, Glycocheck, Microvascular Health Solutions Inc., Salt Lake City, UT, USA) to measure the perfused boundary region (PBR) of sublingual arterial microvessels (5 to 25 μm) an indirect index of endothelial glycocalyx thickness. A higher PBR indicates deeper penetration of erythrocytes into glycocalyx, signifying a reduction in glycocalyx thickness. This method is fast (3 minutes), operator-skill independent, and endorsed by the European Society of Cardiology for assessing endothelial integrity [14].

Arterial Stiffness

Carotid-to-femoral pulse wave velocity (PWV) was estimated using the Complior system (Complior, Alam Medical, Vincennes, France). PWV was calculated as the ratio of the distance between carotid and femoral pulse sites to pulse wave transit time (m/s). Intra-observer variability was 6%.

Echocardiography

Studies were conducted with a Vivid E95 ultrasound system, all studies were digitally stored in a computerized station (Echopac 204 GE, Horten, Norway) and analyses were performed by two observers blinded to clinical and lab data. LV dimensions, volumes, systolic and diastolic properties, LV mass, relative wall thickness (RWT), and left atrial (LA) volume indexed to body surface area were assessed.

Doppler Echocardiography

We measured early mitral inflow (E wave), late A wave and deceleration time (DT) of E mitral wave by Pulsed-wave Doppler. Tissue Doppler imaging recorded myocardial velocities. The sample volume was placed in the septal and lateral sites of the mitral annulus in the apical 4-chamber view to record the LV systolic velocity (S’) and early diastolic velocity (E’). The average value of the velocities at the 2 annular sites was used and the E/E' ratio was calculated.

Coronary Flow Reserve

Coronary flow reserve (CFR) was measured by transthoracic Doppler echocardiography, assessing color-guided pulse-wave Doppler signals in the distal left anterior descending artery. CFR was quantified as the ratio of hyperemic to resting maximal diastolic velocity following adenosine administration.

LV Myocardial Deformation

We measured longitudinal systolic strain (LS) from standard 2-dimensional acquisitions (frame rate: 70-80/sec) with the use of a dedicated software (EchoPac 204 PC, GE Healthcare). Global longitudinal strain (GLS) was calculated using the 17 LV segment model imaged from apical chamber views (4, 2 and 3 chamber view), as previously published [15]. The myocardial deformations at the basal, mid-ventricular and apical segments were averaged to GLS. Inter- and intra-observer variabilities of 8% and 5%, respectively.

**Serum Biomarkers in vivo and in patients**

Serum biomarkers were determined using commercial kits. Malondialdehyde (MDA) (Oxford Biomedical Research, Rochester Hills, Mich, colorimetric assay for lipid peroxidation) was used both for in vivo experiments and for the patient sera. C-Reactive Protein (CRP) (Human CRP ELISA Kit, Cat Number: E-EL-H0043), Proprotein Convertase Subtilisin/Kexin Type 9 (PCSK9) (Human PCSK9 ELISA Kit, Cat Number: E-EL-H1579), 3- Nitrotyrosine (3-NT) (3-NT ELISA Kit, Cat Number: E-EL-0040) and Lipoprotein-a (LP-a) (Human LP-a ELISA Kit, Cat Number: E-EL-H0160), Oxidized Low-Density Lipoprotein (OxLDL) (Human OxLDL ELISA Kit, Cat Number: E-EL-H6021) and (Mouse OxLDL ELISA kit, Cat Number E-EL_M0066) were determined using commercially available ELISA kits purchased by Elabscience (Supplier Bioinnotech) according to manufacturer’s instructions [16].

**Supplementary Tables**

**Supplementary Table 1.** Left Ventricle echocardiographic evaluation *in vivo*.

| *Vehicle (DMSO 5%) group only* | *Baseline* | *8 weeks* | *14 weeks* |
| --- | --- | --- | --- |
|  | ***n=5*** | ***n=5*** | ***n=5*** |
| *HR* | *608.40±16.76* | *548.20±12.87** | *554.00±16.39** |
| *LVEDD(mm)* | *3.37±0.12* | *3.74±0.13** | *3.61±0.02* |
| *LVESD(mm)* | *1.73±0.06* | *2.08±0.14** | *1.97±0.03* |
| *PWTd (mm)* | *0.79±0.01* | *0.77±0.01* | *0.77±0.01* |
| *PWTs (mm)* | *1.31±0.01* | *1.29±0.01** | *1.27±0.01** |
| *FS%* | *48.61±0.40* | *44.55±1.94** | *45.39±0.61* |
| *EF%* | *86.41±0.32* | *82.69±1.96* | *83.69±0.54* |
| *r/h* | *2.12±0.08* | *2.42±0.13** | *2.32±0.05* |

*HR: Heart Rate; LVEDd: Left Ventricular End-Diastolic Diameter, LVESD: Left Ventricular End-Systolic diameter, PWTd: Posterior Wall Thickness at diastole, PWTs: Posterior Wall Thickness at systole; FS: Fractional Shortening; EF: Ejection Fraction, r/h:* *LV radius to PWT ratio. One-way ANOVA, Tukey post hoc test. *p<0.05 vs Baseline.* *Values are presented as Mean ± SEM (n=5).*

**Supplementary Table 2.** Systolic, Mean and Diastolic arterial blood pressure, as determined at three timepoints: Baseline, 8th week before treatments and 14th week after treatments.

|  |  | *Veh  (NS)* | *OL* | *HT* | *Veh  (DMSO 5%)* | *OC* | *OA* |
| --- | --- | --- | --- | --- | --- | --- | --- |
| *Systolic Blood Pressure* | *Baseline* | *97.7 ± 1.2* | *96.8 ± 2.5* | *96.9 ± 2.1* | *98.1±2,1* | *101.8± 1,8* | *99,9 ± 2,9* |
|  | *8 weeks* | *102.3 ± 1.9* | *95.6 ± 3.1* | *96.5 ± 2.7* | *91.9±2.2* | *96.0 ± 3.9* | *91.0 ± 2.6* |
|  | *14 weeks* | *101.0 ± 2.8* | *103.2 ± 3.1* | *100.4 ± 3.4* | *98.3±1.9* | *103.2 ± 4.6* | *99.7 ± 1.6* |
| *Mean  Blood Pressure* | *Baseline* | *84.2 ± 2.2* | *84.5 ± 2.5* | *85.3 ± 2.3* | *81.8 ± 2.0* | *85.7 ±2.7* | *85.9 ± 1.9* |
|  | *8 weeks* | *85.2 ± 1.7* | *79.5 ± 2.9* | *81.4 ± 1.7* | *76.5 ±2.3* | *79.7 ± 2.5* | *75.7 ± 2.4* |
|  | *14 weeks* | *85.6 ± 2.7* | *88.1 ± 3.0* | *85.7 ± 3.3* | *83.8 ± 2.1* | *84.7 ± 3.9* | *86.4 ± 3.0* |
| *Diastolic Blood Pressure* | *Baseline* | *78.3 ± 3.3* | *79.2 ± 2.0* | *80.0 ± 2.4* | *76.7 ± 0.7* | *77.0 ± 3.1* | *81.4 ± 3.2* |
|  | *8 weeks* | *76.7 ± 1.8* | *71.8 ± 2.8* | *74.1 ± 1.5* | *70.3 ± 3.0* | *72.2 ± 2.2* | *73.0 ± 4.5* |
|  | *14 weeks* | *79.5 ± 2.6* | *81.0 ± 3.0* | *78.7 ± 3.2* | *77.5 ± 1.6* | *77.0 ± 3.8* | *83.4 ± 5.4* |

*Two-way ANOVA, Tukey post hoc test separately for Veh (NS), OL, HT and Veh (DMSO 5%), OC, OA. All values are presented as Mean ± SEM (n=5-7).*

**Supplementary Table 3.** Left ventricle echocardiographic evaluation after treatments at the 14th week.

| *14 weeks* | *Veh (DMSO 5%)* | *OL* | *HT* | *OC* | *OA* |
| --- | --- | --- | --- | --- | --- |
|  | ***n=5*** | ***n=5*** | ***n=4*** | ***n=5*** | ***n=5*** |
| *HR* | *554.00±16.39* | *583.25±29.83* | *593.00±34.66* | *593.25±24.06* | *610.40±11.46* |
| *LVEDD(mm)* | *3.61±0.02* | *3.80±0.11* | *3.85±0.19* | *3.65±0.24* | *3.62±0.10* |
| *LVESD(mm)* | *1.97±0.03* | *2.04±0.06* | *2.15±0.13* | *2.11±0.19* | *1.96±0.06* |
| *PWTd (mm)* | *0.77±0.01* | *0.77±0.01* | *0.78±0.01* | *0.79±0.01* | *0.79±0.01* |
| *PWTs (mm)* | *1.27±0.01* | *1.28±0.01* | *1.29±0.01* | *1.28±0.01* | *1.30±0.01* |
| *FS%* | *45.39±0.61** | *46.27±0.77** | *44.24±1.12* | *42.52±1.58* | *45.80±0.58** |
| *EF%* | *83.69±0.54** | *84.46±0.67** | *82.60±1.03* | *80.87±1.52* | *84.06±0.53** |
| *r/h* | *2.32±0.05* | *2.48±0.09* | *2.45±0.13* | *2.31±0.19* | *2.28±0.09* |

*HR: Heart Rate; LVEDd: Left Ventricular End-Diastolic Diameter, LVESD: Left Ventricular End-Systolic diameter, PWTd: Posterior Wall Thickness at diastole, PWTs: Posterior Wall Thickness at systole; FS: Fractional Shortening; EF: Ejection Fraction, r/h: LV radius to PWT ratio. One-way ANOVA, Bonferroni post-hoc test. *p<0.05 vs OC. All values are presented as Mean ± SEM (n=5). LV: Left Ventricle; OL: Oleuropein; HT: Hydroxytyrosol; OC: Oleocanthal; OA: Oleanolic Acid*

**Supplementary Table 4.** Constituents of each combinatorial treatment.

|  | *OL  (20.6 mg/kg)* | *HT  (5.9 mg/kg)* | *OC  (11.6 mg/kg)* | *OA  (17.4 mg/kg)* |
| --- | --- | --- | --- | --- |
| *Combo 1* | √ | √ | - | √ |
| *Combo 2* | √ | √ | √ | - |
| *Combo 3* | √ | √ | √ | √ |

**Supplementary Table 5.** Results from the characteristic peaks in ^1^Η-ΝΜR spectra of OL (600 MHz, MeOD).

| **1H position** | ***δ*_Η_, multiplicity, *J* (Hz), number of H** | ***Chemical structure*** |
| --- | --- | --- |
| 1a’ | 4.22, m, 1H |  |
| 1b’ | 4.12, m, 1H |  |
| 2’ | 2.78, t (7.17), 2H |  |
| 4’ | 6.68, d (2.01), 1H |  |
| 7’ | 6.70, d (8.07), 1H |  |
| 8’ | 6.56, dd (8.01/2.0), 1H |  |
| 1 | 5.93, s, 1H |  |
| 3 | 7.53, s, 1H |  |
| 5 | 3.42, t (8.56), 1H |  |
| 6b | 2.72, dd (13.92/4.64), 1H |  |
| 6a | 2.42, dd (13.98/4.81), 1H |  |
| 8 | 6.10, q (7.10), 1H |  |
| 10 | 1.68, dd (7.17/1.39), 3H |  |
| 12 | 3.73, s, 3H |  |
| Anomeric proton | 4.82, d (7.68), 1H |  |
| Glucose protons | 3.99, q (4.67), 1H |  |
|  | 3.90, dd (11.4/1.61) 1H |  |
|  | 3.69, dd (12.26/5.69), 1H |  |

**Supplementary Table 6.** Results from the characteristic peaks in ^1^Η-ΝΜR spectra of OL (600 MHz, MeOD).

| **1H position** | ***δ*_Η_, multiplicity, *J* (Hz), number of H** | ***Chemical structure*** |
| --- | --- | --- |
| 3 | 3.22, dd (11.4/4.47), 1H |  |
| 12 | 5.28, t (3.2), 1H |  |
| 18 | 2.82, dd (14.19/3.80), 1H |  |
| 23 | 1.25, s, 3H |  |
| 24 | 0.98, s, 3H |  |
| 25 | 0.77, s, 3H |  |
| 26 | 0.75, s, 3H |  |
| 27 | 1.13, s, 3H |  |
| 29 | 0.93, s, 3H |  |
| 30 | 0.91, s, 3H |  |

**Supplementary Table 7.** Left ventricle echocardiographic assessment at 14 weeks after treatment with the selected combinations.

| 14 weeks | Veh  (DMSO 5%) | Combo 1 | Combo 2 | Combo 3 |
| --- | --- | --- | --- | --- |
|  | **n=5** | **n=6** | **n=7** | **n=7** |
| HR | 589.00±13.14 | 558.66±12.17 | 562.57±19.74 | 568.16±14.62 |
| LVEDD(mm) | 3.41±0.17 | 3.68±0.15 | 3.49±0.11 | 3.58±0.16 |
| LVESD(mm) | 1.84±0.10 | 2.07±0.08 | 1.91±0.06 | 1.98±0.10 |
| PWTd (mm) | 0.77±0.01 | 0.78±0.01 | 0.78±0.01 | 0.79±0.01 |
| PWTs (mm) | 1.27±0.01 | 1.25±0.01 | 1.28±0.01 | 1.30±0.01* |
| FS% | 46.16±0.44 | 43.72±0.58* | 45.31±0.52 | 44.70±0.94 |
| EF% | 84.38±0.38 | 82.15±0.56* | 83.61±0.48 | 83.02±0.85 |
| r/h | 2.23±0.13 | 2.35±0.11 | 2.22±0.08 | 2.25±0.11 |

*HR: Heart Rate; LVEDd: Left Ventricular End-Diastolic Diameter, LVESD: Left Ventricular End-Systolic diameter, PWTd: Posterior Wall Thickness at diastole, PWTs: Posterior Wall Thickness at systole; FS: Fractional Shortening; EF: Ejection Fraction, r/h: LV radius to PWT ratio. One-way ANOVA, Bonferroni post-hoc test. *p<0.05 vs Veh (DMSO 5%). All values are presented as Mean ± SEM (n=5-7).*

**Supplementary Table 8.** Hydroxytyrosol at both studied doses and Oleocanthal at the higher dose inhibit NETs formation in PMA- stimulated neutrophils.

| Samples | % NETosis | % Inhibition of NETosis |
| --- | --- | --- |
| Resting | 12.43 ± 8.32 |  |
| Activated | 87.97 ± 10.00 |  |
| Hydroxytyrosol (50μg/ml) | 8.64 ± 3.19 |  |
| Hydroxytyrosol Activated (50μg/ml) | 11.75 ± 1.04 | 93.11 ± 2.20** |
| Hydroxytyrosol Activated (25μg/ml) | 27.55 ± 3.95 | 85.06 ± 8.90** |
| Oleuropein (50μg/ml) | 9.87 ± 1.77 |  |
| Oleuropein Activated (50μg/ml) | 54.88 ± 8.54 | 39.64 ± 25.26 |
| Oleuropein Activated (25μg/ml) | 77.72 ± 10.80 | 20.58 ± 13.44 |
| Oleocanthal (50μg/ml) | 11.81 ± 3.10 |  |
| Oleocanthal Activated (50μg/ml) | 24.59 ± 8.33 | 76.67 ± 11.84* |
| Oleocanthal Activated (25μg/ml) | 74.71 ± 9.14 | 21.16 ± 14.28 |
| Combination (50μg/ml) | 13.18 ± 2.86 |  |
| Combination Activated (50μg/ml) | 54.97 ± 9.46 | 38.20 ± 22.85 |
| Combination Activated (25μg/ml) | 76.10 ± 6.76 | 22.78 ± 8.96 |

*One-way ANOVA, Dunnett's multiple comparisons test. *p<0.05, **p<0.01 compared with Activated.*

**Supplementary Table 9.** Assessment of toxicity markers and signs after 4 weeks of treatment.

|  | Veh  (DMSO 5%) | Combo 2 LD | p value  vs Veh (DMSO 5%) | Combo 2 HD | p value  vs Veh (DMSO 5%) |
| --- | --- | --- | --- | --- | --- |
| Toxicity markers in serum |  |  |  |  |  |
| AST (U/L) | 44.97 ± 5.59 | 42.935 ± 4.28 | 0.9411 | 43.49 ± 4.43 | 0.9656 |
| ALT (U/L) | 18.02 ± 1.92 | 17.52 ± 1.06 | 0.9649 | 17.18 ± 1.64 | 0.9032 |
| Creatinine (mg/dl) | 0.258 ± 0.020 | 0.228 ± 0.020 | 0.4392 | 0.264 ± 0.016 | 0.9663 |
| Urea (mg/dl) | 48.87 ± 1.67 | 50.60 ± 3.33 | 0.8336 | 53.76 ± 1.93 | 0.2851 |
| ALP (U/L) | 62.18 ± 4.45 | 63.44 ± 3.43 | 0.9615 | 66.28 ± 3.63 | 0.6779 |
| LDH (U/L) | 77.99 ± 18.62 | 189.20 ± 26.50 | 0.9971 | 188.38 ± 27.33 | 0.9988 |
| Fasting Glucose (mg/dl) | 71.25 ± 2.13 | 72.14 ± 2.90 | 0.9519 | 72.43 ± 2.44 | 0.9782 |
| Body weight (g) | 27.11 ± 0.43 | 26.40 ± 0.95 | 0.6709 | 25.89 ± 0.48 | 0.3404 |
| Hematological parameters |  |  |  |  |  |
| White Blood Cells (x10^3^/μL) | 8.47 ± 0.51 | 7.99 ± 0.50 | 0.7978 | 7.46 ± 0.77 | 0.4085 |
| Red Blood Cells (x10^6^/μL) | 8.13 ± 0.24 | 8.54 ± 0.27 | 0.3974 | 7.92 ± 0.21 | 0.7665 |
| Hemoglobin (g/dl) | 10.39 ± 0.30 | 10.83 ± 0.32 | 0.5038 | 10.14 ± 0.30 | 0.8018 |
| Hematocrit (%) | 44.01 ± 1.33 | 45.73 ± 1.43 | 0.5519 | 42.77 ± 1.07 | 0.7226 |
| Platelets (x10^3^/μL) | 1023 ± 62.59 | 1171 ± 43 | 0.1186 | 1118 ± 48 | 0.3883 |

*AST: Aspartate Aminotransferase; ALT: Alanine Aminotransferase; ALP: Alkaline Phosphatase; LDH: Lactic Dehydrogenase. One-way ANOVA, Dunnett’s post hoc test. All values are presented as Mean ± SEM (n=6-7).*

**Supplementary Table 10.** *Demographic and clinical data of the study population.*

| Age (years) | 60.2 ± 8.9 |
| --- | --- |
| Sex (male) | 4/15 (26.6%) |
| Heart rate (bpm) | 64.8 ± 5.8 |
| Systolic Blood Pressure (mmHg) | 127.4 ± 23.4 |
| Diastolic Blood Pressure (mmHg) | 71.3 ± 12.9 |
| Surgical Treatment n, (%) | 2/15 (13.3%) |
| Percutaneous Treatment n, (%) | 12/15 (80%) |
| 1 Vessel disease n, (%) | 6/15 (40%) |
| 2 Vessels disease n, (%) | 6/15 (40%) |
| 3 Vessels Disease n, (%) | 3/15 (20%) |
| Left Main Disease n, (%) | 2/15 (13.3%) |
| Statins n, (%) | 15/15 (100%) |
| B- blocker n, (%) | 15/15 (100%) |
| Αngiotensin-converting enzyme inhibitor/ angiotensin receptor n, (%) | 15/15 (100%) |
| Calcium Channel Blockers n, (%) | 5/15 (33.3%) |
| Diuretics n, (%) | 3/15 (20%) |
| Acetylsalicylic acid n, (%) | 14/15 (93.3%) |
| Clopidogrel n, (%) | 7/15 (46.6%) |
| Ticagrelor n, (%) | 3/15 (20%) |
| Prasugrel n, (%) | 1/15 (6,6%) |
| Direct Oral Anticoagulants n, (%) | 2/30 (13.3%) |
|  |  |

**Figure Legends**

**Supplementary Fig. 1.** **Compounds’ structure verification after isolation and purity determination.** ^1^H NMR spectra of A. isolated Oleuropein (MeOD), B. Hydroxytyrosol (MeOD), C. Oleocanthal (CDCl_3_), D. Oleanolic Acid (CDCl_3_) (600 MHz, Bruker) and Ε. Superimposed HPLC-DAD chromatograms of the isolated Oleocanthal, Oleuropein and Hydroxytyrosol monitored under 280 nm.

**Supplementary Fig. 2**. **Graphical representation of the in vivo experimental protocols.** For each experimental protocol a timeline is depicted with the main procedures and the primary endpoint. Combo: Combinatorial therapy; DMSO: Dimethyl sulfoxide; HD: High Dose; HT: Hydroxytyrosol; LD: Low Dose OA: Oleanolic Acid; OC: Oleocanthal; OL: Oleuropein.

**Supplementary Fig. 3.** **Power analysis for sample size determination**. Calculation of the minimum number of animals required for the **A.** 1^st^, **B.** 2^nd^ and **C.** 3^rd^ series of experiments.

**Supplementary Fig. 4. Graphical representation of the clinical study design and population.** The study is randomized cross-over, double-blind, placebo-controlled. The evaluation before and after the treatment periods included echocardiographic assessment and blood collection.

**Supplementary Fig. 5.** **Oleuropein, Oleocanthal and Oleanolic Acid reduce the infarct size in healthy mice**. Graphs of **A.** Infarct/Risk Area Ratio % and **B.** Area at risk % of DMSO 5%-, OL-, HT-, OC- and OA- treated mice. A, B One-way ANOVA, Tukey post hoc test. *p<0.05, **p<0.01. All values are presented as Mean ± SEM. DMSO: Dimethyl sulfoxide; HT: Hydroxytyrosol; OA: Oleanolic Acid; OC: Oleocanthal; OL: Oleuropein.

**Supplementary Fig. 6.** **Characterization and validation of the diet-induced MS in wild type mice.** Graphs of **A.** Fasting blood glucose of both NS- and DMSO- treated groups. **B.** Glucose curves of DMSO 5%-treated group and **C.** Calculated AUC for each glucose curve. D. Total cholesterol and Triglycerides in plasma. **E.** Relative adipose tissue weight in the ND group and the NS- and DMSO 5%- treated groups. **F.** Body weight monitored weekly in a group of mice receiving ND and in the two vehicle-treated groups. **G.** Systolic, Mean and Diastolic blood pressure. A, D Two-way ANOVA, Sidak and Tukey post hoc test. C, E, G One way ANOVA, Sidak post hoc test. F. Two-way ANOVA, Tukey post hoc test. ^#^p<0.05, ^###^p<0.001, ^####^p<0.0001 vs Baseline, ^$$^p<0.01, ^$$$$^p<0.0001 vs 8 weeks, ns p>0.05, *p<0.05, ***p<0.001, ****p<0.0001, ^++^p<0.01, ^++++^p<0.0001 ND vs Veh (DMSO 5%), ^ꞎ^p<0.05, ^ꞎꞎ^p<0.01, ^ꞎꞎꞎ^p<0.001, ^ꞎꞎꞎꞎ^p<0.0001 ND vs Veh (NS). All values are presented as Mean ± SEM. DMSO: dimethyl sulfoxide; NS: normal saline

**Supplementary Fig. 7. Structural verification of OL and OA in the treatment solutions of each isolated compound separately and their mixture.** ^1^ H-NMR spectra (600 MHz, MeOD) of OA (green), OL+OA mixture (OL:OA 1:0,84) (red) and OA (blue). OA: Oleanolic Acid; OL: Oleuropein

**Supplementary Fig. 8. Hydroxytyrosol, Oleocanthal and Combination 2 protect LDL particles from oxidation *in vitro* and significantly reduce oxidative stress markers in mice with diet-induced MS. A**) Representative blot at 2 hours of reperfusion and **B)** relative densitometric graph after normalization to total protein via ponceau staining of nitro-tyrosine. Bar plots of **C)** MDA and **D)** oxidized LDL in the plasma of mice with MS at the endpoint of the experimental protocol (n=6-8). **E)** Bar plot of lipid peroxidation products after exposure of LDL to CuSO4 *in vitro*, in the presence of DMSO 5%, OL, HT, OC or Combo 2 (n=4-5 independent experiments). One way ANOVA, Tukey post hoc test, *p<0.05, **p<0.01, ***p<0.001, vs Veh (DMSO 5%). All values are presented as Mean ± SEM. 3-NT: 3-nitrotyrosine; Combo 2: Combinatorial therapy with oleuropein, hydroxytyrosol and oleocanthal; DMSO: Dimethyl sulfoxide; HT: Hydroxytyrosol; LDL: Low density lipoprotein; MDA: malondialdehyde; OC: Oleocanthal; OL: Oleuropein; TBARs: thiobarbituric acid reactive substances.

**Supplementary Fig. 9**. **Histological evaluation of tissue specimens stained with H&E revealed no signs of toxicity after Combo 2 treatment in low and high dose regimens.** Representative images of the normal tissue architecture observed in all groups for A. and B. heart tissue, C. and D. liver, E. and F. kidney, G. and H. pancreas, I. and J. small intestine, K. and L. lung.

**Supplementary Fig. 10. Original Western blots for the proteins presented in Figure 6A of the manuscript.** GAPDH or α-Actinin respectively served as loading control for quantification. Akt: Protein Kinase B; Bax: BCL2 Associated X; Bcl-xL: B-cell lymphoma-extra-large; CAT: Catalase; Combo 2: Combinatorial therapy with oleuropein, hydroxytyrosol and oleocanthal; DMSO: Dimethyl sulfoxide; GAPDH: Glyceraldehyde-3-phosphate dehydrogenase; HO-1: Heme oxygenase 1; HT: Hydroxytyrosol; MnSOD: Manganese-superoxide dismutase; ND: Normal Diet; OC: Oleocanthal; OL: Oleuropein.

**Supplementary Fig. 11. Original Western blots for the proteins presented in Figure 6B of the manuscript.** β-tubulin served as loading control for the quantification. Combo 2: Combinatorial therapy with oleuropein, hydroxytyrosol and oleocanthal; DMSO: Dimethyl sulfoxide; HT: Hydroxytyrosol; NOX -1: NADPH oxidase 1, Nrf2: nuclear factor erythroid 2–related factor 2; OC: Oleocanthal; OL: Oleuropein

**Supplementary Fig. 12. Original Western blots for the 3-NT quantification presented in Supplementary figure 8 6B of the present file.** Each lane was quantified relative to the total protein measured with Ponceau. β. Combo 2: Combinatorial therapy with oleuropein, hydroxytyrosol and oleocanthal; DMSO: Dimethyl sulfoxide; HT: Hydroxytyrosol; OC: Oleocanthal; OL: Oleuropein

**Supplementary Figures**

**Supplementary Fig. 1**

**A.**

**
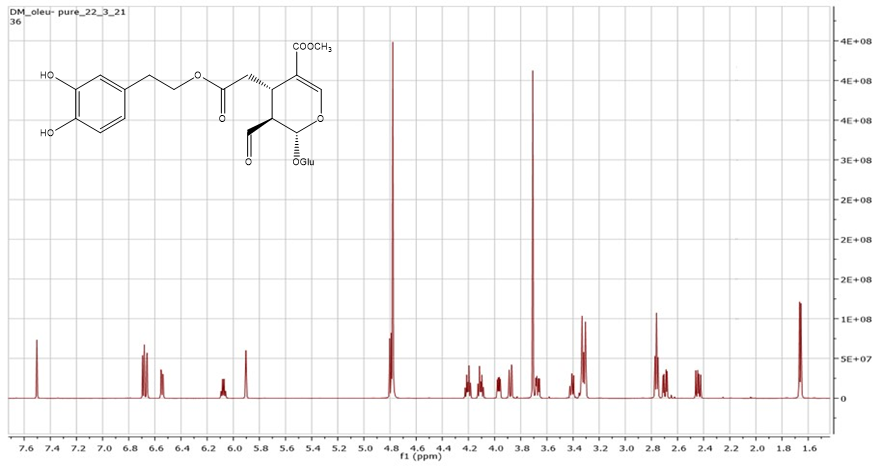
**

**B.**


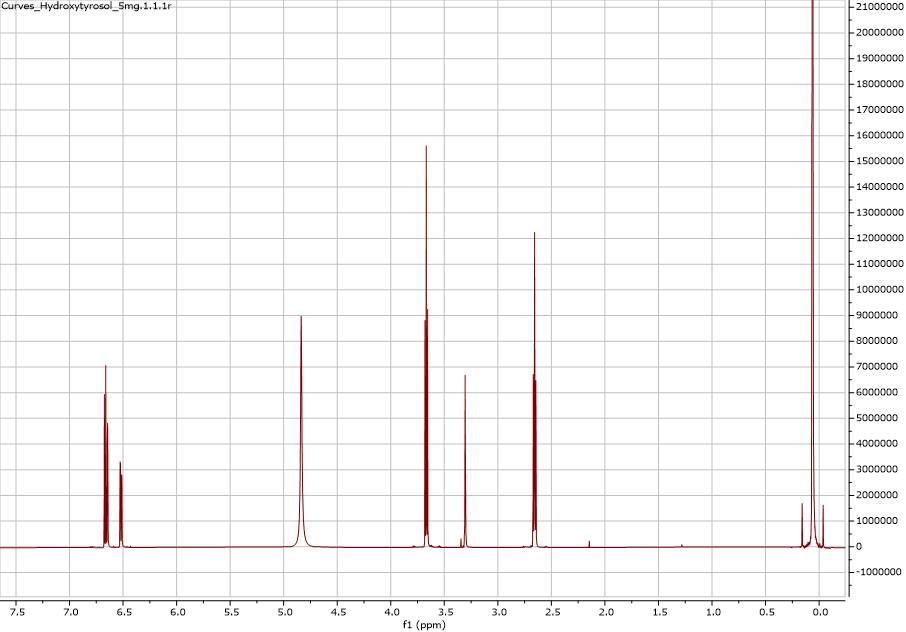


**C.**

**
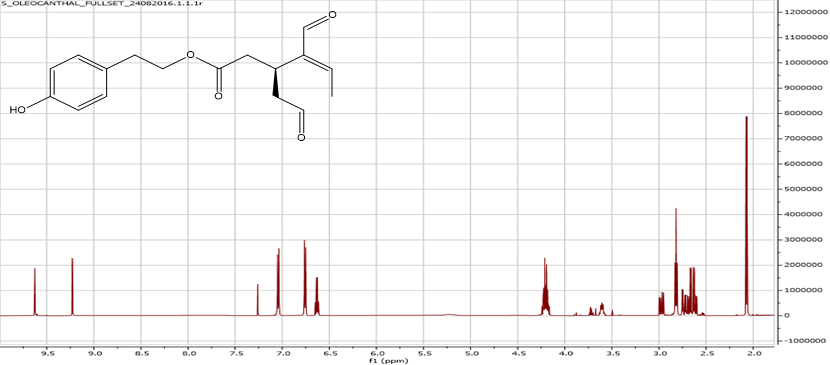
**

**D.**

**
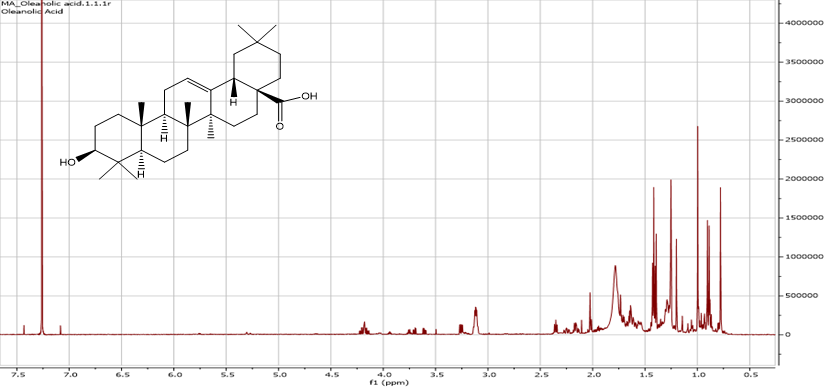
**

**E.**

**
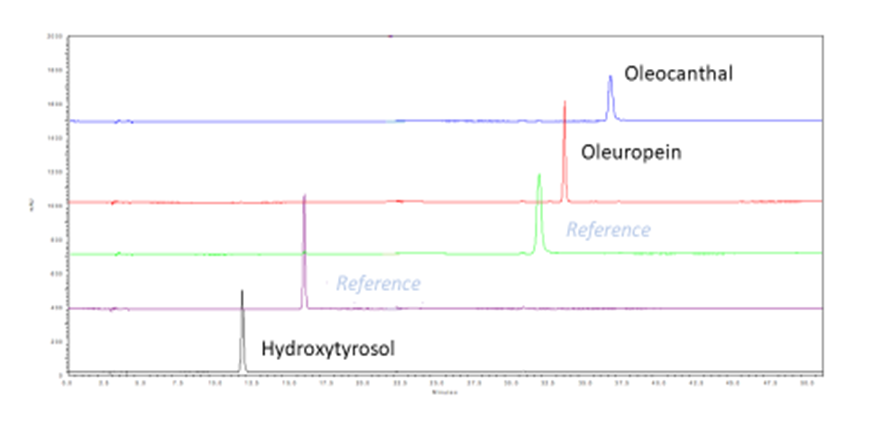
**

**Supplementary Fig. 2**


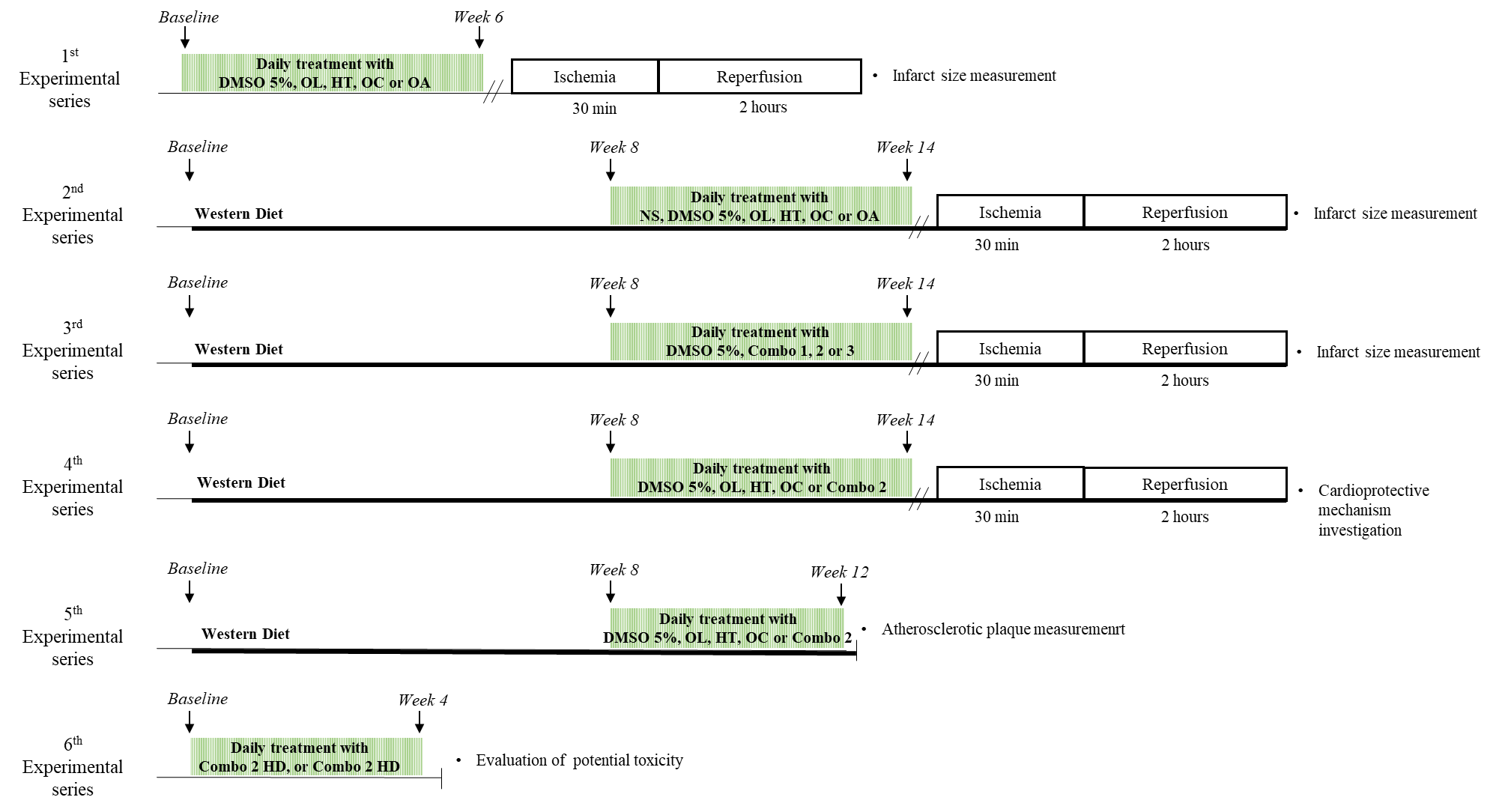


**Supplementary Fig. 3**

A.


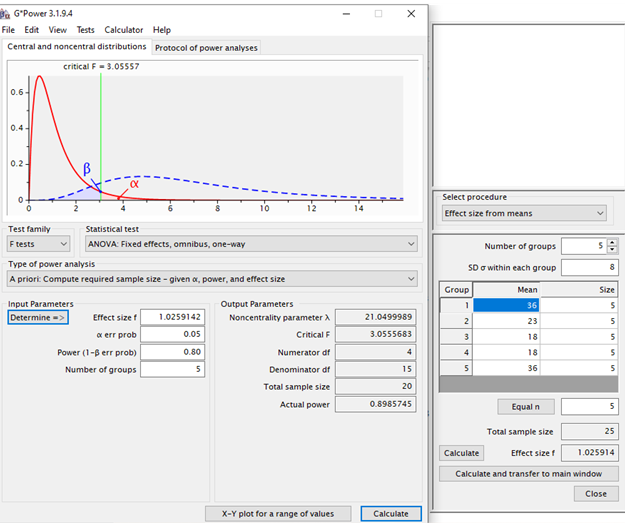


B.


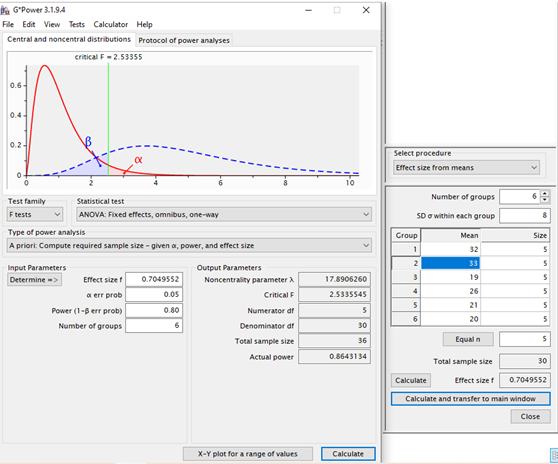


C.


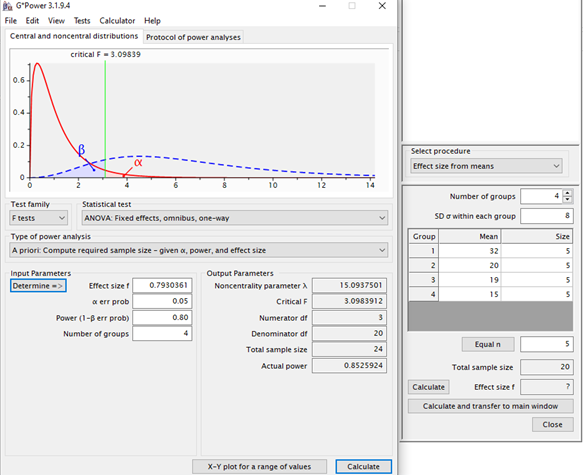


**Supplementary Fig. 4**

*
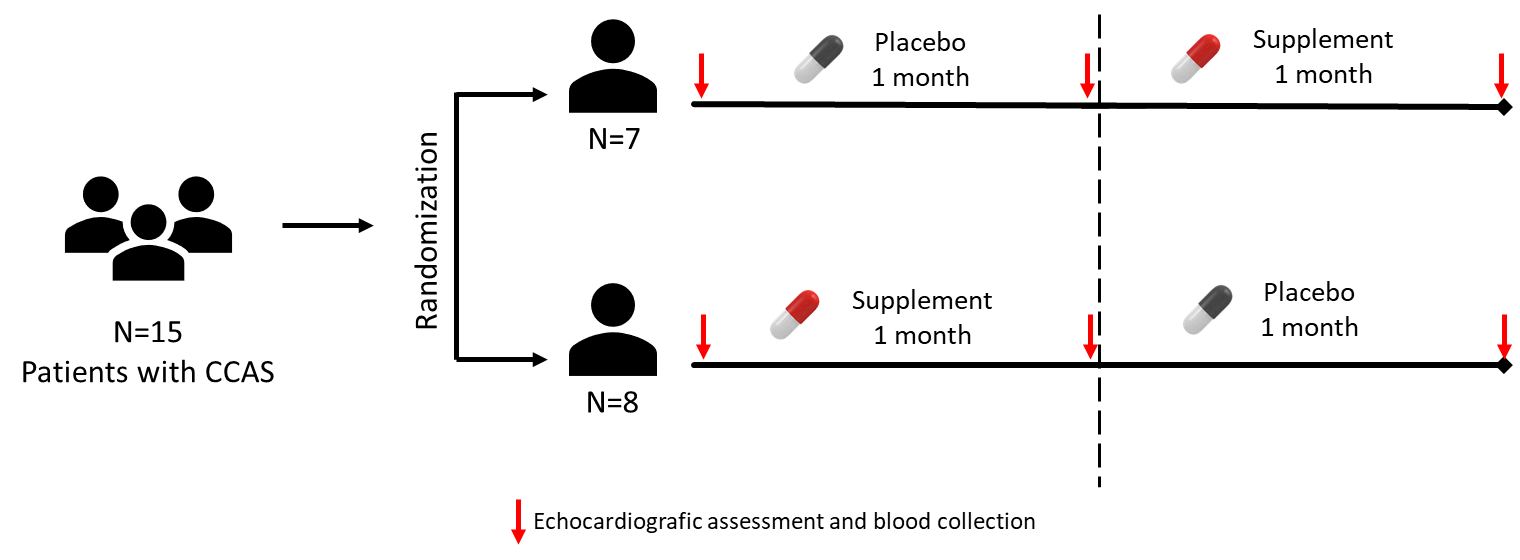
*

**Supplementary Fig. 5**


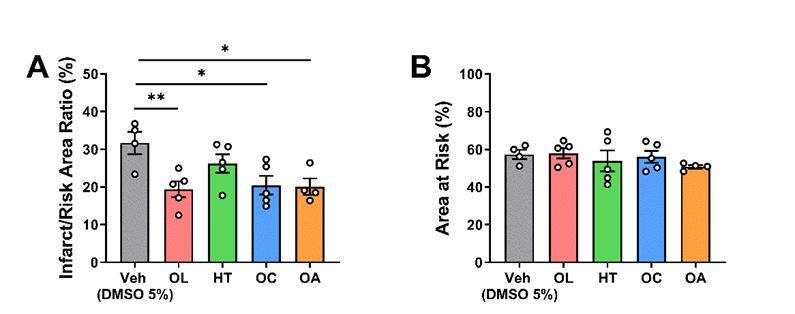


**Supplementary Fig. 6**





**Supplementary Fig. 7**


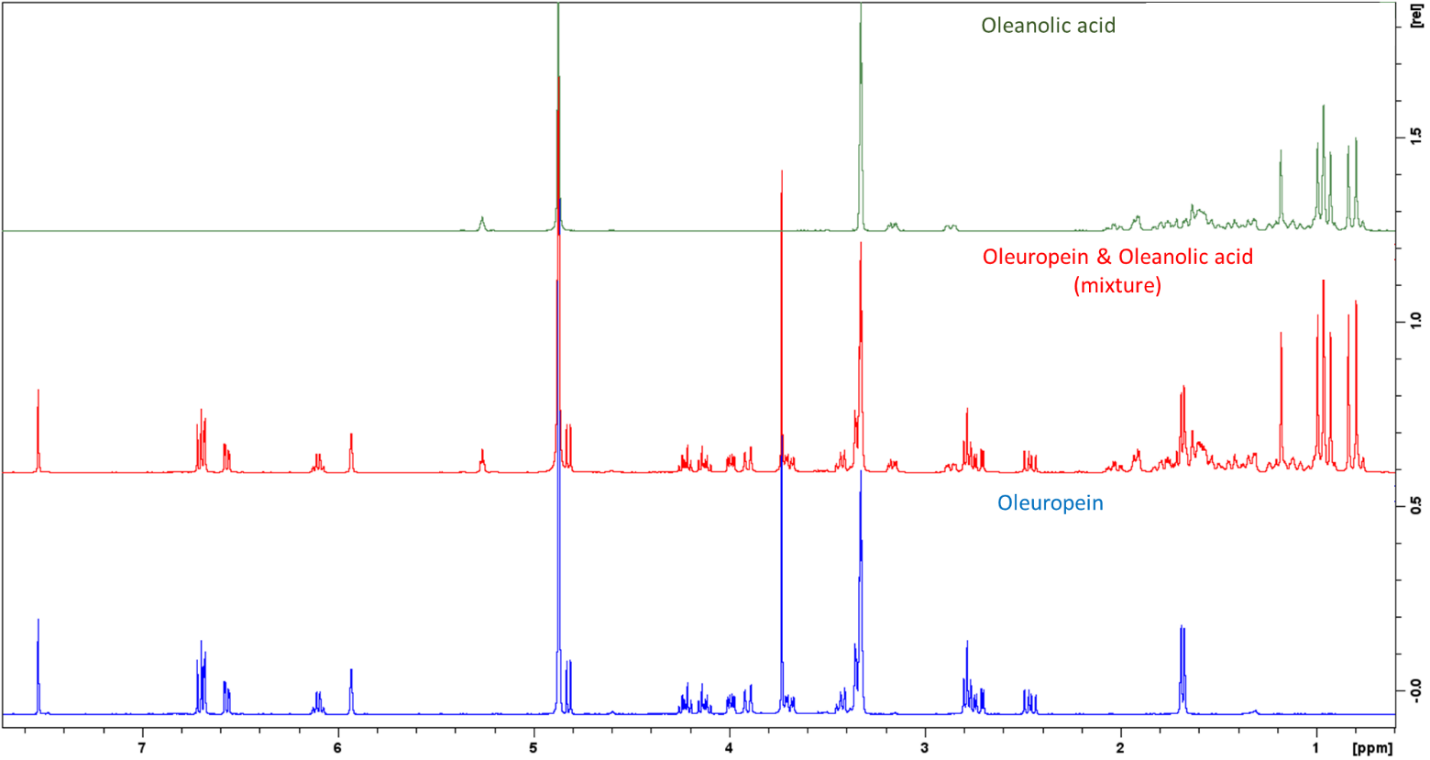


**Supplementary Fig. 8**





**Supplementary Fig. 9**

**
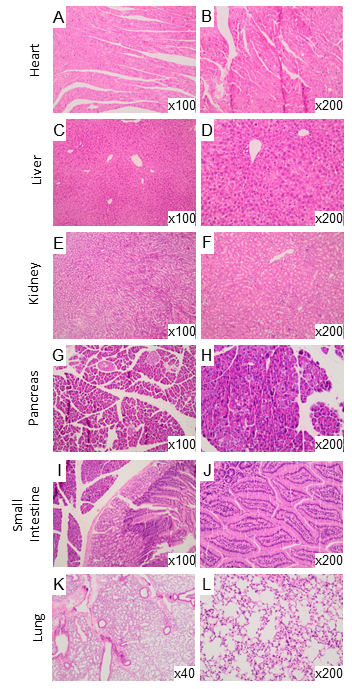
**

**
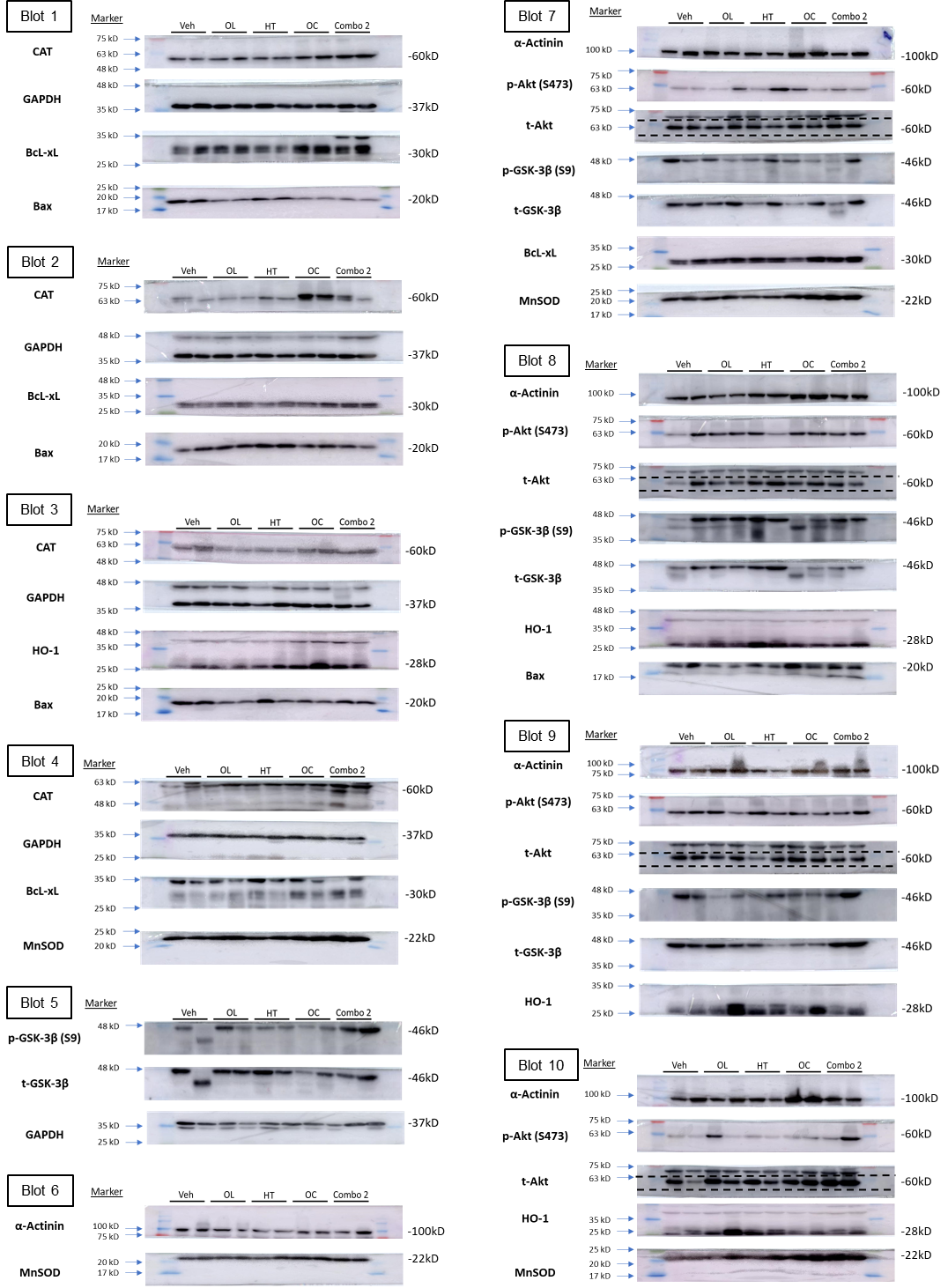
Supplementary Fig.10**


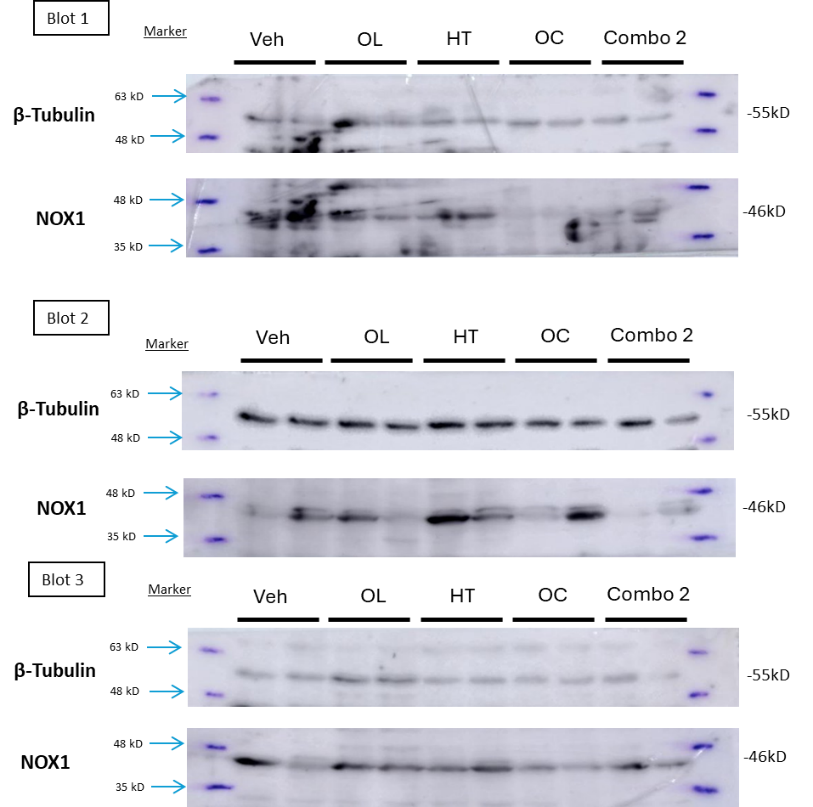

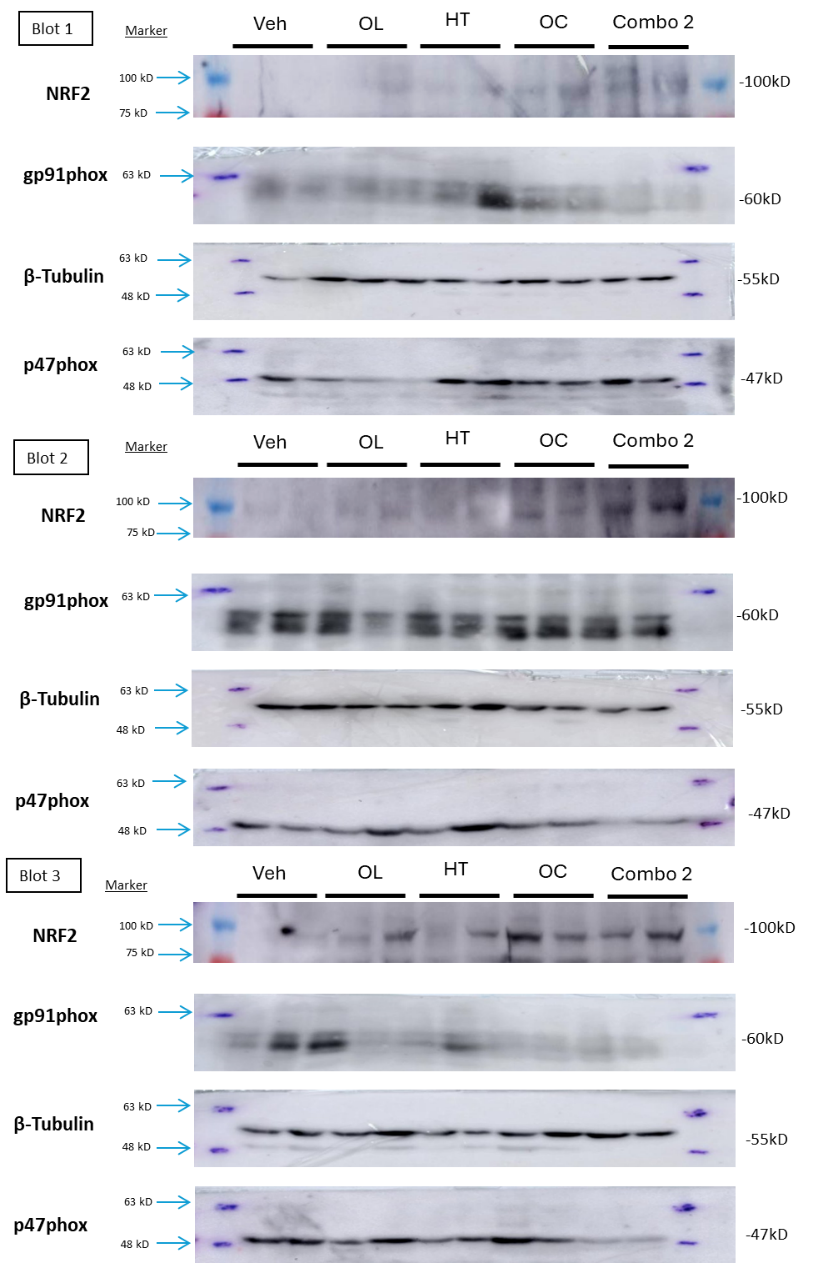
**Supplementary Fig. 11**

**Supplementary Fig. 12**


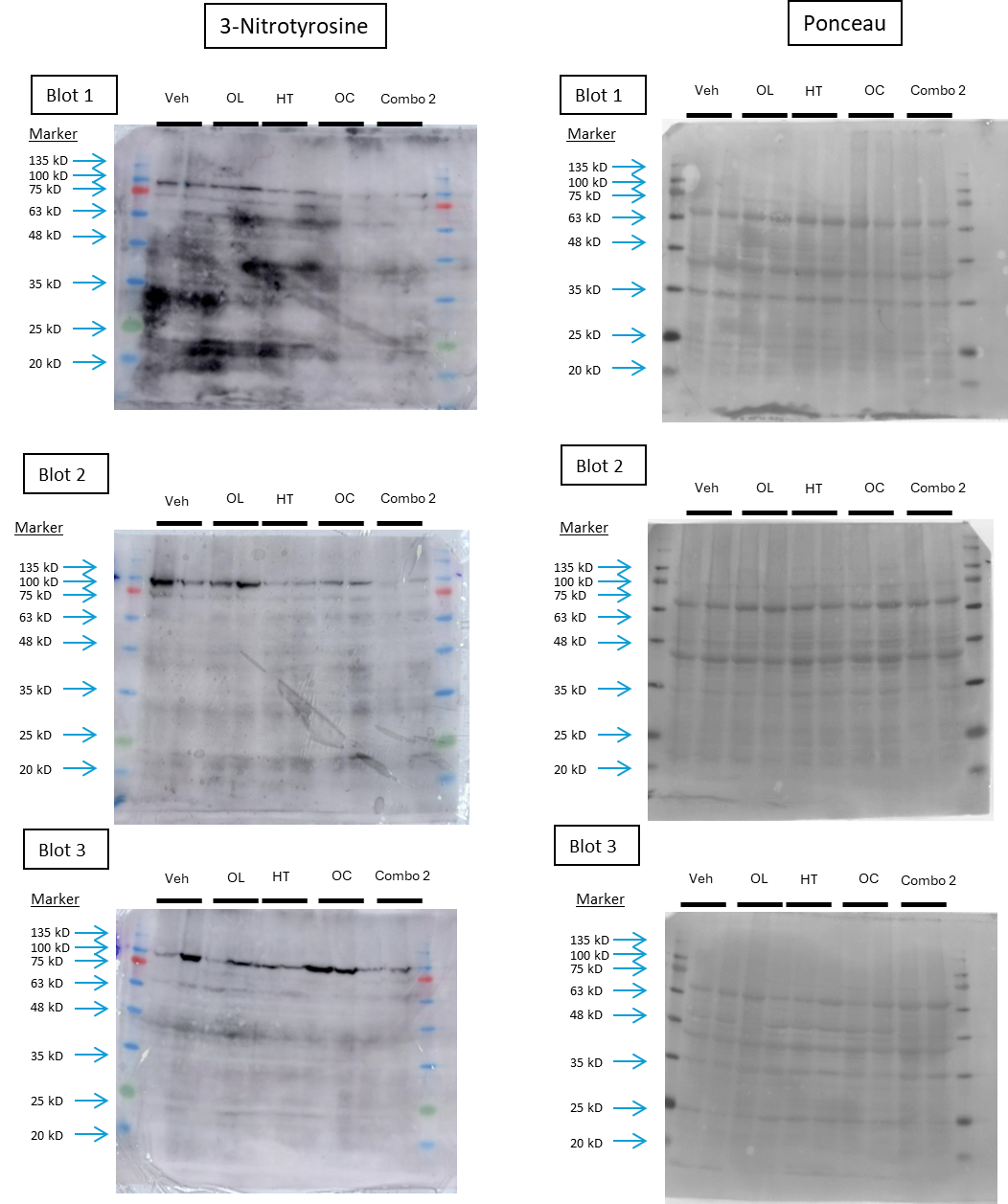


**References**

1. Boka, V.-I.; Argyropoulou, A.; Gikas, E.; Angelis, A.; Aligiannis, N.; Skaltsounis, A.-L. Employment of High-Performance Thin-Layer Chromatography for the Quantification of Oleuropein in Olive Leaves and the Selection of a Suitable Solvent System for Its Isolation with Centrifugal Partition Chromatography. *Planta Med.* **2015**, *81*, 1628–1635, doi:10.1055/s-0035-1558140.

2. EFSA Panel on Dietetic Products, Nutrition and Allergies (NDA); Turck, D.; Bresson, J.-L.; Burlingame, B.; Dean, T.; Fairweather-Tait, S.; Heinonen, M.; Hirsch-Ernst, K.I.; Mangelsdorf, I.; McArdle, H.J.; et al. Safety of Hydroxytyrosol as a Novel Food Pursuant to Regulation (EC) No 258/97. *EFSA J.* **2017**, *15*, e04728, doi:10.2903/j.efsa.2017.4728.

3. Andreadou, I.; Efentakis, P.; Balafas, E.; Togliatto, G.; Davos, C.H.; Varela, A.; Dimitriou, C.A.; Nikolaou, P.-E.; Maratou, E.; Lambadiari, V.; et al. Empagliflozin Limits Myocardial Infarction in Vivo and Cell Death in Vitro: Role of STAT3, Mitochondria, and Redox Aspects. *Front. Physiol.* **2017**, *8*.

4. Andreadou, I.; Iliodromitis, E.K.; Mikros, E.; Constantinou, M.; Agalias, A.; Magiatis, P.; Skaltsounis, A.L.; Kamber, E.; Tsantili-Kakoulidou, A.; Kremastinos, D.T. The Olive Constituent Oleuropein Exhibits Anti-Ischemic, Antioxidative, and Hypolipidemic Effects in Anesthetized Rabbits12. *J. Nutr.* **2006**, *136*, 2213–2219, doi:10.1093/jn/136.8.2213.

5. Pei, Y.; Chen, J.; Xie, L.; Cai, X.; Yang, R.-H.; Wang, X.; Gong, J. Hydroxytyrosol Protects against Myocardial Ischemia/Reperfusion Injury through a PI3K/Akt-Dependent Mechanism. *Mediators Inflamm.* **2016**, *2016*, 1232103, doi:10.1155/2016/1232103.

6. Mapanga, R.F.; Rajamani, U.; Dlamini, N.; Zungu-Edmondson, M.; Kelly-Laubscher, R.; Shafiullah, M.; Wahab, A.; Hasan, M.Y.; Fahim, M.A.; Rondeau, P.; et al. Oleanolic Acid: A Novel Cardioprotective Agent That Blunts Hyperglycemia-Induced Contractile Dysfunction. *PLoS ONE* **2012**, *7*, e47322, doi:10.1371/journal.pone.0047322.

7. Nikolaou, P.E.; Efentakis, P.; Abu Qourah, F.; Femminò, S.; Makridakis, M.; Kanaki, Z.; Varela, A.; Tsoumani, M.; Davos, C.H.; Dimitriou, C.A.; et al. Chronic Empagliflozin Treatment Reduces Myocardial Infarct Size in Nondiabetic Mice Through STAT-3-Mediated Protection on Microvascular Endothelial Cells and Reduction of Oxidative Stress. *Antioxid. Redox Signal.* **2021**, *34*, 551–571, doi:10.1089/ars.2019.7923.

8. Benedé-Ubieto, R.; Estévez-Vázquez, O.; Ramadori, P.; Cubero, F.J.; Nevzorova, Y.A. Guidelines and Considerations for Metabolic Tolerance Tests in Mice. *Diabetes Metab. Syndr. Obes. Targets Ther.* **2020**, *13*, 439–450, doi:10.2147/DMSO.S234665.

9. Andrikopoulos, S.; Blair, A.R.; Deluca, N.; Fam, B.C.; Proietto, J. Evaluating the Glucose Tolerance Test in Mice. *Am. J. Physiol.-Endocrinol. Metab.* **2008**, *295*, E1323–E1332, doi:10.1152/ajpendo.90617.2008.

10. Nikolaou, P.E.; Mylonas, N.; Makridakis, M.; Makrecka-Kuka, M.; Iliou, A.; Zerikiotis, S.; Efentakis, P.; Kampoukos, S.; Kostomitsopoulos, N.; Vilskersts, R.; et al. Cardioprotection by Selective SGLT-2 Inhibitors in a Non-Diabetic Mouse Model of Myocardial Ischemia/Reperfusion Injury: A Class or a Drug Effect? *Basic Res. Cardiol.* **2022**, *117*, 27, doi:10.1007/s00395-022-00934-7.

11. Tsoumani, M.; Georgoulis, A.; Nikolaou, P.-E.; Kostopoulos, I.V.; Dermintzoglou, T.; Papatheodorou, I.; Zoga, A.; Efentakis, P.; Konstantinou, M.; Gikas, E.; et al. Acute Administration of the Olive Constituent, Oleuropein, Combined with Ischemic Postconditioning Increases Myocardial Protection by Modulating Oxidative Defense. *Free Radic. Biol. Med.* **2021**, *166*, 18–32, doi:10.1016/j.freeradbiomed.2021.02.011.

12. Lambadiari, V.; Thymis, J.; Kouretas, D.; Skaperda, Z.; Tekos, F.; Kousathana, F.; Kountouri, A.; Balampanis, K.; Parissis, J.; Andreadou, I.; et al. Effects of a 12-Month Treatment with Glucagon-like Peptide-1 Receptor Agonists, Sodium-Glucose Cotransporter-2 Inhibitors, and Their Combination on Oxidant and Antioxidant Biomarkers in Patients with Type 2 Diabetes. *Antioxidants* **2021**, *10*, 1379, doi:10.3390/antiox10091379.

13. Ikonomidis, I.; Tzortzis, S.; Andreadou, I.; Paraskevaidis, I.; Katseli, C.; Katsimbri, P.; Pavlidis, G.; Parissis, J.; Kremastinos, D.; Anastasiou-Nana, M.; et al. Increased Benefit of Interleukin-1 Inhibition on Vascular Function, Myocardial Deformation, and Twisting in Patients With Coronary Artery Disease and Coexisting Rheumatoid Arthritis. *Circ. Cardiovasc. Imaging* **2014**, *7*, 619–628, doi:10.1161/CIRCIMAGING.113.001193.

14. Lekakis, J.; Abraham, P.; Balbarini, A.; Blann, A.; Boulanger, C.M.; Cockcroft, J.; Cosentino, F.; Deanfield, J.; Gallino, A.; Ikonomidis, I.; et al. Methods for Evaluating Endothelial Function: A Position Statement from the European Society of Cardiology Working Group on Peripheral Circulation. *Eur. J. Cardiovasc. Prev. Rehabil.* **2011**, *18*, 775–789, doi:10.1177/1741826711398179.

15. Lang, R.M.; Badano, L.P.; Victor, M.-A.; Afilalo, J.; Armstrong, A.; Ernande, L.; Flachskampf, F.; Foster, E.; Goldstein, S.A.; Kuznetsova, T.; et al. Recommendations for Cardiac Chamber Quantification by Echocardiography in Adults: An Update from the American Society of Echocardiography and the European Association of Cardiovascular Imaging. *J. Am. Soc. Echocardiogr.* **2015**, *28*, doi:10.1016/j.echo.2014.10.003.

16. Ikonomidis, I.; Katogiannis, K.; Chania, C.; Iakovis, N.; Tsoumani, M.; Christodoulou, A.; Brinia, E.; Pavlidis, G.; Thymis, J.; Tsilivarakis, D.; et al. Association of Hydroxytyrosol Enriched Olive Oil with Vascular Function in Chronic Coronary Disease. *Eur. J. Clin. Invest.* **2023**, e13983, doi:10.1111/eci.13983.
